# A Decision Aid to Support Shared Decision Making About Mechanical Ventilation in Severe Chronic Obstructive Pulmonary Disease Patients (InformedTogether): Feasibility Study

**DOI:** 10.2196/jopm.9877

**Published:** 2018-05-14

**Authors:** Melissa Basile, Johanna Andrews, Sonia Jacome, Meng Zhang, Andrzej Kozikowski, Negin Hajizadeh

**Affiliations:** ^1^ Department of Medicine Northwell Health Manhasset, NY United States

**Keywords:** Chronic Obstructive Pulmonary Disease, COPD, shared decision making, life support

## Abstract

**Background:**

Severe Chronic Obstructive Pulmonary Disease patients are often unprepared to make decisions about accepting intubation for respiratory failure. We developed a Web-based decision aid, InformedTogether, to facilitate severe Chronic Obstructive Pulmonary Disease patients’ preparation for decision making about whether to accept invasive mechanical ventilation for respiratory failure.

**Objective:**

We describe feasibility testing of the InformedTogether decision aid.

**Methods:**

Mixed methods, pre- and postintervention feasibility study in outpatient pulmonary and geriatric clinics. Clinicians used InformedTogether with severe Chronic Obstructive Pulmonary Disease patients. Patient-participants completed pre- and postassessments about InformedTogether use. The outcomes measured were the following: feasibility/acceptability, communication (Combined Outcome Measure for Risk Communication [COMRADE], Medical Communication Competency Scale [MCCS], Observing Patient Involvement [OPTION] scales), and effectiveness of InformedTogether on changing patients' knowledge, Decisional Conflict Scale, and motivation.

**Results:**

We enrolled 11 clinicians and 38 Chronic Obstructive Pulmonary Disease patients at six sites. Feasibility/acceptability: Clinicians and patients gave positive responses to acceptability questions (mean 74.1/89 max [SD 7.24] and mean 59.63/61 [SD 4.49], respectively). Communication: 96% of clinicians stated InformedTogether improved communication (modified MCCS mean 44.54/49 [SD 2.97]; mean OPTION score 32.03/48 [SD 9.27]; mean COMRADE Satisfaction 4.31/5.0 [SD 0.58]; and COMRADE Confidence 4.18/5.0 [SD 0.56]). Preference: Eighty percent of patients discussed preferences with their surrogates by 1-month. Effectiveness: Knowledge scores increased significantly after using InformedTogether (mean difference 3.61 [SD 3. 44], *P*=.001) and Decisional Conflict decreased (mean difference Decisional Conflict Scale pre/post -13.76 [SD 20.39], *P*=.006). Motivation increased after viewing the decision aid.

**Conclusions:**

InformedTogether supports high-quality communication and shared decision making among Chronic Obstructive Pulmonary Disease patients, clinicians, and surrogates. The increased knowledge and opportunity to deliberate and discuss treatment choices after using InformedTogether should lead to improved decision making at the time of critical illness.

## Introduction

### Background

The lung disease Chronic Obstructive Pulmonary Disease (COPD) develops over time. One-third of mortality in COPD patients is due to progressive respiratory failure and exacerbations [1]. In the event of severe exacerbation patients need to decide whether to accept invasive mechanical ventilator (IMV) support. The IMV–associated risks include the likelihood that patients cannot be extubated, tracheostomy, and admission to a long-term care facility [2,3]. Many patients and surrogates may accept IMV by default [4] without the chance to fully consider the risks and benefits, leading to potentially preference-incongruent decisions [3].

We developed a Web-based decision aid called InformedTogether to facilitate severe COPD patients’ preparation for decision making about whether to accept IMV for respiratory failure (Multimedia Appendix 1). Development and usability testing of InformedTogether is described elsewhere [3,5-7]. InformedTogether was designed for implementation first in an outpatient clinic visit, between COPD patients and clinicians, and then by patients and surrogates. It communicates important information: the likelihood of respiratory failure in patients with severe COPD, treatment options (Full Code versus do not intubate; DNI), risks and benefits of these options, tailored prognostic estimates, and resources for decision making and advance-care-planning including Medical Order for Life-Sustaining Treatment (MOLST) forms [8]. Patients can take notes in comment boxes, and complete preference-elicitation exercises to help consider risks and benefits for each option. In this study we describe the results of feasibility testing of the InformedTogether decision aid in outpatient clinics.

## Methods

We enrolled clinicians, patients, and surrogates in a pre-post feasibility study between April 2016 and September 2016. The clinician participants included the following: pulmonologists, geriatricians, and advance-practice providers (ie, nurse practitioners and respiratory therapists). The patient participants were adults diagnosed with COPD (Forced Expiratory Volume in the first second [FEV1]<50%) who were fluent in either English or Spanish and their surrogates.

### Study Design

We selected the sample size based on other feasibility studies and our experience with recruitment from the outpatient clinics [9]. All participating clinicians first received thirty-minute in-person training on how to navigate the InformedTogether decision aid. Each week a research coordinator searched the electronic health records (EHR) to determine whether COPD patients meeting eligibility were scheduled for outpatient clinic visits or pulmonary rehab during that week. Before approaching the patient, we contacted their pulmonary clinician to determine whether or not there might be a reason why we should exclude that patient from the study. Patients were then approached during their regular outpatient visits. Enrolled patients completed baseline surveys assessing knowledge about COPD treatment choices, decisional conflict about advance-care-planning, and motivation to make an Advance Directive (AD). Patients then met with their clinicians who could choose whether and how much of the decision aid they would use with their patients. In order to determine the feasibility of implementing the decision aid in real-life clinical scenarios, we allowed the clinician to determine what portions of the decision aid were appropriate to share with a particular patient. This included choosing not to use the decision aid if they did not feel that it was the right moment to have an advance care planning discussion. Clinician-patient visits were audio-recorded. Patients were interviewed directly after the clinic visit, re-asked baseline assessment questions, asked about their reactions to the decision aid, acceptability of use and their satisfaction with clinicians’ communication. Patients received Option Grids summarizing information in InformedTogether (Multimedia Appendix 2) [10]. Study materials were available in English and Spanish. All Spanish language documents were translated using a certified medical translator. Clinicians were interviewed after each visit. Patients were additionally interviewed via phone 1-month after the clinic visit, where we measured whether they accessed the decision aid, discussed it, and changed their motivation to make an AD. If patients agreed, we contacted their surrogates to measure surrogates’ reactions to the decision aid, and their conversations about either InformedTogether specifically, or ACP in general with patient participants. Based on initial clinician feedback, we allowed decision aid use testing dedicated advance-care-planning appointments instead of regularly scheduled outpatient visits.

### Primary Outcomes

The primary aim was to determine the feasibility and acceptability of implementing the decision aid in regular outpatient clinic visits. We also sought to assess the quality of the decision aid as measured by changes in knowledge, decisional conflict and motivation to make advance care plans. Secondary outcomes were the effect of the decision aid on communication and changes in decisions that were made over time. The outcomes measured included the following: (1) feasibility and acceptability of implementing InformedTogether in outpatient clinics (ie, questions focused on use of InformedTogether, recommendations to others, trust in content, fit within clinic workflow [Multimedia Appendix 3]); (2) outcomes important for informed decision making [11-15]: improved knowledge, Decisional Conflict Scale (DCS), and motivation to make an AD (ie, 5-point Likert Scale, completing an AD); and (3) quality of communication between clinicians and patients (ie, Combined Outcome Measure for Risk Communication [COMRADE], Medical Communication Competency Scale [MCCS], and OPTION scales [16-18]), and between patients and surrogates (ie, 1-month follow-up interviews).

### Data Analysis

Descriptive statistics summarized the results to close-ended questions. Kappa statistics with 95% confidence interval were calculated for the degree of agreement between pre-post responses in knowledge, DCS and motivation to make an AD. Univariable analyses were used to explore associations between variables. For example, the Two-Sample *t* test/Wilcoxon Rank Sum Test or the analysis of variance (ANOVA)/Kruskal-Wallis test was used for: (1) relationships between baseline demographics (analyzed as categorical variables), and (2) pre-post changes in the total scores (analyzed as continuous variables). The Spearman correlation coefficient measured the association between outcomes (ie, whether a change in the total score of knowledge was associated with a change in motivation score). To test whether there was a trend in level of shared decision making (OPTION scale) over time per clinician, we used a Linear Mixed Effects model.

All clinician-patient encounters and 1-month follow up interviews were audio recorded and transcribed verbatim. Spanish language audios were transcribed by a certified translator. We analyzed transcripts from the patients’ clinic visits and open-ended responses using qualitative methods. Three members of the research team read all transcripts, and developed a list of themes inductively (ie, allowing ideas to develop organically through reading the transcripts), and deductively (ie, hypothesis-driven and related to our outcomes, as well as to our theory that the impact of non-biomedical knowledge including prior lived experiences may impact a patient’s ability to understand medical information or apply it to themselves when making decisions about their care). After developing the final set of themes, we developed a codebook consisting of the themes names; definitions; sample text; and inclusion and exclusion criteria. Using the codebook, two researchers (MB and AK) coded the transcripts using NVivo. Coding comparison performed on 10% of transcripts (n=7) showed 99.8% agreement and a Cohen kappa of 0.67, indicating good agreement [19]. Discrepancies were discussed between the principal investigator and the lead qualitative researcher to reach a consensus.

### Declarations

#### Ethics, Consent, and Permissions

The study was approved by the Northwell Health Institutional Review Board and we obtained written informed consent from all participants.

#### Consent for Publication

This article does not contain individual patient data.

#### Availability of Data and Materials

All data sets are available from the corresponding author.

## Results

In this study, we enrolled: 11 clinicians and 38 patients (after approaching 70 eligible patients) with severe COPD. Of these, 28 spoke English while 10 spoke Spanish. The study was conducted at 5 Pulmonary clinics (n=10 or 90% of the total participants) and 1 Geriatric clinic (n=1 or 10% of the total participants). A total of 38 clinician-patient encounters using InformedTogether were recorded. One-month after the clinic visit, we interviewed 30 patient participants (8 of the original study participants were lost to follow-up) and 7 surrogate caregivers of these participants (Table 1 and Multimedia Appendix 4).

### Feasibility and Acceptability of Implementation

The clinician participants used 78%, with a mean of 21 minutes per patient, of InformedTogether in most visits. They preferred using the decision aid during separate advance-care-planning visits instead of during the regularly scheduled clinic visits. Clinicians gave strongly positive responses to acceptability questions with a mean 74.1 out of 89 maximum points (SD 7.2). Clinicians indicated that they found the images and diagrams depicting intubation and tracheostomy and the prognostic estimates to be particularly useful in communicating with their patients. Those who only used select portions of the decision aid focused on those pages.

The patient participants gave strongly positive responses to acceptability questions in 95% of the cases with a mean score 59.6 out of 61 (SD 4.5) indicating that they would highly recommend it to others. In fact, 80% stated they would definitely recommend it. They indicated a high degree of trust in the decision aid content, with 80.9% (SD 17.0) using a scale 0-100%, measured based on the following question: “How sure are you that the estimates given in the decision aid are correct?” (Table 2).

### Communication

The clinician participants stated that InformedTogether improved their communication with a mean score 44.5 out of 45 maximum score (SD 3.0) on the MCCS. InformedTogether facilitated shared decision making based on a mean OPTION score of 32.0 out of 40 (SD 9.3). Statistically there was no significant difference in Option score between clinicians (Kruskal-Wallis Test χ^2^=16.2; *P*=.06). However, with each clinician’s additional use of InformedTogether there was a statistically significant OPTION score increase by 1.9 points (SD 0.5, *P*=.001) based on the Linear Mixed Effects Model.

The patient participants expressed a high degree of satisfaction with clinicians’ communication with a mean COMRADE Satisfaction with Communication 4.3 out of 5.0 (SD 0.6) with COMRADE Confidence in Decision 4.2 out of 5.0 (SD 0.6). At 1-month follow-up, 80% of participants stated they had discussed the decision aid with their surrogates (Table 3).

**Table 1:** Basic demographics of patient participants (N=38).

Patient Characteristics		Results
Age, mean (SD)		66.6 (10.0)
**Marital status, n (%)**		
	Single	6 (15.8)
	Married	15 (39.5)
	Divorced/separated	7 (18.4)
	Widowed	8 (21.1)
	Other	2 (5.3)
**Gender, n (%)**		
	Female	19 (50.0)
**Race/ethnicity, n (%)**		
	White	19 (50.0)
	Black/African American	7 (18.4)
	Hispanic/Latino** **	11 (29.0)
	Other	1 (2.6)
**Religious affiliation, n (%)**		
	Catholic	26 (68.4)
	Jewish	3 (7.9)
	None	3 (7. 9)
	Other	6 (15.8)
**Employment status, n (%)**		
	Employed full-time	4 (10.5)
	Retired	23 (60.5)
	Unemployed	11 (29.0)
**Highest level of education, n (%)**		
	Less than grade 9	4 (10.5)
	Grade 9 to 12	17 (44.7)
	Some college or higher	17 (44.7)
**Economic class, n (%)**		
	Lower class	7 (18.4)
	Lower-middle class	5 (13.2)
	Middle class	24 (63.2)
	Upper-middle class or higher	2 (5.3)
**Born in the US, n (%)**		
	Yes	32 (84.2)
**Years Living in US if foreign-born**		
	Mean (SD)	24 (16.3)
**Language(s) spoken at home, n (%)**		
	English	33 (86.8)
	Spanish	9 (23.7)

**Table 2:** Feasibility and acceptability of implementation.

Feasibility & acceptability	Mean score (SD)
Clinician rated feasibility and acceptability (maximum score 89)	74.1 (7.2)
Patient rated feasibility and acceptability (maximum score 61)	59.6 (4.5)
Trust in the decision aid (how sure are you that the estimates given are correct; maximum score 100)	80.9 (17.0)

**Table 3:** Clinician-patient communication using the InformedTogether decision aid. MCCS: Medical Communication Competency Scale; OPTION: Observing Patient Involvement Scale; COMRADE: Combined Outcome Measure for Risk Communication.

Communication	Mean (SD)
Clinician self-rated Communication (modified MCCS, maximum score 49)	44.5 (3.0)
Observer-rated Shared Decision Making (OPTION scale, maximum score 48)	32.0 (9.3)
Difference in OPTION score with each additional use	1.9 (0.5)^a^
Difference in OPTION score between clinicians	16.2 (0.06)^b^
Patient-rating of satisfaction with communication (COMRADE sub-scale, maximum score 5)	4.3 (0.6)
Patient-rating of confidence in decision (COMRADE sub-scale, maximum score 5)	4.2 (0.6)

^a^*P*=.008; Linear Mixed Effects Model.

^b^Kruskal-Wallis Test.

### Knowledge, Decisional Conflict, and Motivation

Overall, the participants in our study had improved knowledge, motivation to make an AD, and decreased decisional conflict after using the InformedTogether decision aid (Table 4).

Seven patients expressed emotional discomfort while viewing the decision aid (Multimedia Appendix 5). For these patients, their sense of discomfort ranged from feeling that the information was too much for them to handle at that moment; or surprise because they had never had an end of life or advance care planning discussion before. For others, there was sadness when recalling prior experiences with family members on life support or sadness thinking about how their families would react to seeing them intubated. Most of the 7 participants expressing discomfort rated their health as fair or better and had never thought about the need to make a decision about life supporting treatments. Despite this, all of these 7 patient participants stated they would recommend that the decision aid be used with other patients (mean 3.6 out of 4, SD 0.5). One participant stated, “It made me uncomfortable but I would still recommend it”. Among our 38 patient-participants, only 1 person asked to stop using the decision aid and this was due to her expressed discomfort at the information contained in the decision aid. One clinician chose not use the decision aid with a patient who had been diagnosed with lung cancer just after agreeing to participate in the study. The clinician did not feel it was appropriate to have an advance care planning conversation on the same day as she was going to give the patient this diagnosis.

At 1-month, 8 patient participants were lost to follow-up. Of these 2 were lost due to death and 1 due to hospitalization. Notably, 5 of these 8 participants had expressed discomfort viewing the decision aid. Twenty of the 30 patients (67%) interviewed at 1-month follow-up had accessed InformedTogether after the clinic visit, rating it as moderately easy to use with a mean (SD) of 6.4 (3.0) using a 1-10 scale.

Seven surrogate decision makers were interviewed 1-month after the clinic visit, and of these, 5 stated that they had seen the decision aid. All surrogate participants stated that the decision aid was informative and very easy to use (9.5 [SD 0.6]). Several mentioned that it helped them to understand their family member’s condition (COPD) better; and that using it led to “more discussion”, “in-depth discussion” about what their family member would want. Notably, two surrogates were spouses of participants who died during the 1-month time period. Both stated that the information contained in the decision aid factored into their spouses’ decision not to be intubated:

She had thought about it at that time and we […] discussed […] those charts you had given us with the percentages on it and the more you looked, it didn’t look like a very bright future because of the percentiles that had come in so far on these tests and I think that she just felt that she just wanted to be at ease with herself and she was ready to make peace […] I think there was a couple of questions that maybe we were thinking about going a different way–maybe going on a breathing tube for a period of time but then again, we didn’t know what the period of time would have been and what the end result would have been. I think coming out of the meeting and then going home and […] discussing it again with our children, we came to the conclusion that she felt what she really wanted to do [to decline intubation].

**Table 4:** Effectiveness of InformedTogether decision aid on improving patients' knowledge of Chronic Obstructive Pulmonary Disease and motivation to engage in advance care planning, and reducing decisional conflict. DCS: Decisional Conflict Scale.

Effectiveness		Mean (SD)	*P* value^a^
**Knowledge (maximum score 18)**			
	Pre-InformedTogether knowledge	8.2 (3.4)	
	Post-InformedTogether knowledge	11.8 (3.0)	
	Increase in knowledge	3.6 (3.4)^b^	*P*=.001
	Number of participants with increased knowledge	32 (84.2%)	
	Number of participants with no change in knowledge	2 (5.3%)	
	Number of participants with decreased knowledge	4 (10.5%)	
**DCS (maximum score 80)**			
	Pre-InformedTogether DCS	31.3 (25.1)	
	Post-InformedTogether DCS	17.9 (15.9)	
	Decrease in DCS	-13.8 (20.4)^b^	*P*=.006
	Number of participants with increased DCS	3 (14.3%)	
	Number of participants with no change in DCS	3 (14.3%)	
	Number of participants with decreased DCS	15 (71.4%)	
**Motivation (maximum score 5)**			
	Pre-InformedTogether motivation	4.3 (1.0)	
	Post-InformedTogether motivation	4.4 (0.7)	
	Post-InformedTogether motivation at 1-month	4.6 (0.8)	
	Increase in motivation	0.1 (0.8)^b^	*P*=.54
	Increase in motivation at 1-month	0.3 (1.1)^b^	*P*=.10
	Number of participants with increased motivation	6 (17.1%)	
	Number of participants with no change in motivation	23 (65.7%)	
	Number of participants with decreased motivation	6 (17.1%)	

^a^*P* value shown when Wilcoxon signed rank test is performed.

^b^Wilcoxon signed rank test.

### Effect on Change in Decision and Discussions About “Trial Intubation”

At baseline, among participants who had previously thought about whether to accept intubation, 10 chose Full Code, 5 chose DNI, and 6 were unsure. After InformedTogether, 11 chose Full Code, 5 chose DNI and 5 participants were unsure. At 1 month, all participants were asked about their preferred choice: 20 participants chose Full Code, and 8 chose DNI. Five participants stated their decision had changed after viewing the decision aid. For example, 3 from DNI to Full Code, 1 from Full Code to trial intubation, and 1 from Full Code to DNI. For an example of one patient’s progression of the AD decision from baseline to the 1-month follow-up, see Multimedia Appendix 6.

In 13 clinician-patient encounters the information presented about tracheostomy (for patients who cannot be extubated) led to a discussion about ‘trial intubation’ as an additional decision point not formally presented in the decision aid. For many, this was prompted by the patient (Multimedia Appendix 6). Some patients initially brought up trial intubation with their clinicians during their initial use of Informed Together. Other patients initially stated during their discussions with their clinicians that they would not want intubation, but, during the one month follow up interview, they expressed an interest in trial intubation. Several of these patients asked if it would be possible to put a specific length of time for a trial in their ADs. At 1-month, 9 participants stated that they would choose ‘trial intubation’ after seeing InformedTogether. Among the 13 people who brought up the topic of trial intubation, 8 of them had slightly lower DCS scores after seeing InformedTogether, (16.8 out of 64). Among the 22 who stated they had previously thought about whether to accept IMV, 9 of them did not discuss trial intubation with their clinicians while viewing the decision aid. On average, decisional conflict scores were slightly higher among this group for an average DCS 19.6 out of 64 compared to the group who had discussed trial intubation with their clinician (Table 5).

**Table 5:** Change in decision. DNI: do not intubate.

Change in Decision	Stated preference by number
Pre-InformedTogether decision in those who had thought about intubation before viewing InformedTogether	10 Full Code; 5 DNI; 6 unsure
Post-InformedTogether decision in those who had thought about intubation before viewing InformedTogether	11 Full Code; 5 DNI; 5 unsure
Direction of change in decision among 'Full Code' at baseline	8 stayed Full Code, 1 DNI, 1 unsure
Direction of change in decision among 'DNI' at baseline	2 stayed DNI, 2 Full Code, 1 unsure
Direction of change in decision among 'unsure' at baseline	4 stayed unsure and 2 DNI

### Univariable Analysis Results

See Multimedia Appendices 7-Multimedia Appendices 9)

In exploring associations between patient factors and outcomes, we found changes in knowledge were greater in participants with lower education levels (9.1; *P*=.05), and lower QOL (6.1; *P*=.02). There was a smaller decrease in DCS score in those expressing a religious affiliation (3.9; *P*=.05). There was a higher level of decisional conflict after viewing the decision aid in those with a lower QOL (6.0; *P*=.02). There were significant associations between change in motivation at 1-month follow-up and QOL (6.3; *P*=.01), and a history of frequent hospitalizations (5.1; *P*=.02).

In exploring associations between outcomes, we found a negative correlation between satisfaction with communication and DCS (Spearman Correlation Coefficient -0.5 for Satisfaction with Communication Scores 11-20 [*P*=.005]), indicating that those expressing higher satisfaction with clinician communication had lower DCS after viewing the decision aid. Additionally, there was a trend suggesting that increased knowledge was associated with decreased DCS (Spearman Correlation Coefficient -0.4 [*P*=.08]).

## Discussion

### Principle Results

We found that it is feasible to implement InformedTogether in outpatient clinics. InformedTogether was acceptable to users, supported high-quality communication, and shared decision making between clinicians and patients, and patients and surrogates. Half of participants who did not have a decision at baseline, had made one at 1-month follow-up. This included both those participants who indicated that they had made an AD within 1 month of viewing the decision aid, and those who had conversations with their family members regarding their preferences – several of whom had shown the decision aid to their family members, but who did not formalize their preferences in an AD. Decisions made after using InformedTogether were more fully informed, as indicated by increased knowledge and changes in the decision over time. InformedTogether was also effective in prompting conversations between patients and surrogates. Poignantly, surrogates for the 2 participants who died during the study period stated InformedTogether had facilitated decision making at the time a decision became necessary.

Most participants stated they would choose intubation over DNI. Qualitative analysis suggests this may be due to discussions about ‘trial intubation’. Although tracheostomy was only discussed in the context of patients who cannot be extubated, it was correctly seen as a separate decision point where individuals can decide to stop IMV and move to comfort-only measures. We speculate that this may lead to reduced decisional conflict for those people who may be comforted by the fact that they can revisit their decision to be intubated (ie, they can try intubation and decide to be removed if they choose). While this represents practice in many ICUs and serves as an attempt to reduce uncertainty about outcomes, it remains unclear what the optimal trial timeframe should be, which is an important area of future investigation.

InformedTogether communicates different treatment options and estimated prognosis, and guides patients through preference elicitation exercises to help identify and communicate outcomes which are most important to patients. A common criticism of ADs is that they may not be applicable to different situations and that surrogate decision makers would benefit more from understanding general preferences for outcomes over single-scenario treatment decisions [20]. The recent shift to advance-care-planning as opposed to documenting an AD emphasizes discussions about choices and preferred outcomes over time [21]. InformedTogether facilitates these iterative discussions and deliberation for both patients and their surrogates.

We measured change in knowledge, motivation to make a decision, and decisional conflict as indicators of informed decision making [11-13,22] and found improvements in each of these outcomes after using InformedTogether. However, DCS increased in three patients. In conventional representations of informed decision making, as knowledge increases decisional conflict should decrease [22]. Decisional conflict may increase with knowledge, particularly for decisions with a high degree of uncertainty about outcomes [23,24]. This increase may therefore represent a necessary step in deliberation, and with time, decisional conflict could in fact, decrease.

Discomfort with the discussion is known to be a barrier to clinicians initiating advance-care-planning conversations with patients [25]. The use of sensitive language within InformedTogether helped clinician-patient conversations, and patient participants strongly recommended the decision aid for use with other patients. We found that among those clinicians who used InformedTogether multiple times during the study, there was an increase in OPTION score with each use. This suggests that over time, as clinicians became more comfortable using the decision aid, their ability to engage in high quality shared decision-making conversations with their patients improved. This includes the extent to which the clinician involves that patient in decision making, ensures that the patient understands what the decision to be made involves, makes that patient aware that there is more than one choice for their clinical problem, and clearly explains the pros and cons of each choice, all of which are measured through the validated OPTION scale. These findings support the use of InformedTogether to facilitate shared advance-care-planning conversations partly because of the patient-centered language that clinicians can either read or adapt over time to tailor conversations as needed.

### Limitations

An important limitation of our study is that we were not able to follow participants beyond one month and therefore have limited data to suggest effectiveness of the decision aid on actual decision making at the time of exacerbation. We also do not have a comparison of relative effectiveness compared to other forms of information communication and/or standard care. Although an increase in knowledge and the opportunity to engage with surrogate decision makers about treatment choices and preferences will likely lead to more informed decision making during exacerbation, it remains to be seen whether this in fact leads to more preference-congruent care and satisfaction with an actual decision. Ultimately, these are the outcomes that InformedTogether is designed to improve and will be the focus of future studies.

Notably, we were unable to contact 5 of the 38 patients who were interviewed for 1-month follow up. It is very possible that this subgroup of patients had different opinions about the decision aid’s feasibility and their use of the decision aid with their family after the clinic visit. Our inability to assess this is a limitation of the study and inherent in the risk of loss to follow up within many clinical studies.

A further limitation is that we did not have a large enough sample size to draw conclusions about potential associations between patient factors and outcomes found in univariate analysis. These are; however, hypothesis generating and will be further explored in a larger future trial.

### Comparison with Previous Work

We tested the feasibility and effectiveness of implementing a decision aid (InformedTogether) which includes personalized prognostic estimates for patients with severe COPD, in outpatient pulmonary and geriatric clinics. InformedTogether includes a prognostic model which is tailored based on a patients’ age, and estimates both in-hospital survival, discharge to nursing home versus home, rehospitalization and 12-month survival.

We are the first group to test the feasibility of communicating personalized prognostic estimates to inform advance care planning in outpatient clinics for patients with severe COPD, and to test the feasibility of translating comparative effectiveness research results into accessible and usable formats for shared decision making. InformedTogether was tested in both English and Spanish languages and found to be acceptable to both patients and clinicians, as well as the surrogate decision makers of patients. In addition to finding that InformedTogether was well received by participants, we also found it to effectively change knowledge, decisional conflict and motivation to make advance care plans for individual patients. Importantly, InformedTogether promoted high quality communication and shared decision making between clinicians and their patients, and clinicians used the information and language within the decision aid to personalize the information and to guide their patients’ decision making.

### Conclusion

Our study demonstrates that the InformedTogether decision aid facilitates high quality communication between clinicians and their severe COPD patients about treatment choices and likely outcomes in the event of acute respiratory failure. The improved knowledge, reduced decisional conflict and increased motivation seen as a result of using InformedTogether should support patients and their families to make more informed decisions about whether to accept life supporting technologies in the event of critical illness. Iterative discussions using decision aids such as ours, which include patient-centered communication about tailored prognostic estimates and treatment choices, can facilitate deliberation and communication about treatment choices so that patients and families are more prepared to make preference-congruent decisions about life-supporting technologies. Preparation for decision making about life supporting technologies is particularly important for patients with advanced chronic diseases who are at highest risk for complications, and patients and families need to be informed about the possible short and long-term consequences of their treatment choices. Clinicians can support this informed decision making by initiating conversations about advance care plans, and decision aids such as InformedTogether can overcome several barriers to initiating these discussions.

## Appendix

Multimedia Appendix 1 Screen Shots of The InformedTogether Decision Aid.

Multimedia Appendix 2 InformedTogether Option Grid for a COPD Patient Age Range 66-70 Years Old.

Multimedia Appendix 3 Feasibility and Acceptability Questionnaires.

Multimedia Appendix 4 Additional Demographic Characteristics.

Multimedia Appendix 5 Distress experienced while viewing decision aid.

Multimedia Appendix 6 Patient Interest in Trial Intubation.

Multimedia Appendix 7 Univariable Analysis Associations between Communication and Outcomes.

Multimedia Appendix 8 Univariable Analysis Associations between Outcomes and Patient Demographics.

Multimedia Appendix 9 Univariable Analysis Associations between Outcomes and Patient Self-Rated Health.

## Abbreviations

AD: advance directive
COMRADE: Combined Outcome Measure for Risk Communication and Treatment Decision Making Effectiveness
COPD: Chronic Obstructive Pulmonary Disease
DC: decisional conflict
DCS: Decisional Conflict Scale
DNI: do not intubate
HER: electronic health records
IMV: invasive mechanical ventilation
MCCS: Medical Communication Competency Scale
MOLST: medical order for life-sustaining treatment
OPTION scale: Observing Patient Involvement scale
