# Supplementary material for: A Decision Aid to Support Shared Decision Making About Mechanical Ventilation in Severe Chronic Obstructive Pulmonary Disease Patients (InformedTogether): Feasibility Study
Source: J Particip Med. 2018 May 14;10(2):e7. doi: 10.2196/jopm.9877 (PMC7251980; doi:10.2196/jopm.9877)
Supplement: Multimedia Appendix 1 [file jopm_v10i2e7_app1.pdf]

Supplementary Web-only Content

Basile M, et al; InformedTogether: A decision aid to support shared decision making about mechanical ventilation in severe COPD patients.

Supplementary Figure 1: InformedTogether Option Grid for a COPD Patient Age Range 66-70 Years Old

Supplementary Table 1 Additional Demographic Characteristics
Supplementary Table 2 Distress Experienced While Viewing Decision Aid
Supplementary Table 3 Patient Interest in Trial Intubation
Supplementary Table 4 Univariable Analysis: Associations between Communication and Outcomes
Supplementary Table 5 Univariable Analysis: Associations between Outcomes and Patient Demographics
Supplementary Table 6 Univariable Analysis: Associations between Outcomes and Patient Self-rated Health

Supplementary Figure 2: Screen Shots of The InformedTogether Decision Aid
Supplementary Figure 3a: Feasibility and Acceptability Questionnaires for Patients
Supplementary Figure 3b: Feasibility and Acceptability Questionnaires for Clinicians

This supplementary material has been provided by the authors to give readers additional information about their work.

Supplementary Figure 1: InformedTogether Option Grid for a COPD Patient Age Range 66-70 Years Old

Here are some answers to questions you may have when choosing between a Breathing Machine versus Comfort Measures Only (palliative care) when you have a severe COPD exacerbation and can't breathe.

Frequently asked questions	**Breathing Machine After A Severe Exacerbation**	**No Breathing Machine After A Severe Exacerbation (Comfort Care Only)**
**Tell me more about each of my options.**	A breathing tube goes into a patient's mouth and into their windpipe. This tube is attached to a breathing machine which blows air into the airway. This is called **intubation/mechanical ventilation and** is one kind of life support.	Comfort Care Only means a patient has chosen NOT to have any life support machines. In this case patients are treated with pain medications and other treatments to make them as comfortable as possible. This is also called palliative/hospice care.
**When are these treatment options offered to me?**	When you are not able to breathe and other treatments in the hospital (such as oxygen, inhalers, steroids and mask ventilation/bipap) do not work.	
**Will it help me to live longer?**	A breathing machine cannot cure COPD, but it may enable you to live longer by helping you breathe when you cannot do so on your own.	Most people who choose NOT to have any life support will not survive, but instead will have the care focused on a peaceful death.
**Will it be painful?**	A breathing machine can cause problems and make you uncomfortable. Doctors will give you pain medications and medications to help with this discomfort.	With Comfort Care, all treatments are focused on pain relief and medicines are given so you don't feel like you are having a hard time breathing.
**What are the benefits?**	• You may live longer.<br>• Breathing is usually easier because a machine will be doing the breathing for you.	• You will be given medication to treat pain, trouble breathing and help you to feel relaxed.<br>• The medications will support a peaceful death
**What are the risks?**	Although patients may live longer**,**<br>• 20-30% of patients may not be able to come off the breathing machine and **will not be able to return back home**.<br>• 100% of patients **cannot talk** while on the breathing machine.<br>• 100% of patients **cannot eat** while on the breathing machine.<br>• **Almost 100%** cannot walk because they are attached to the breathing machine.	• Most patients who choose <u>not</u> to be treated with a breathing machine and choose comfort measures only, **will not survive**.
**What are the <u>possible</u> results?**	**On average,**<br>• About 73 out of 100 people will survive in the hospital.<br>• About 37 of the 73 survivors will be discharged to a nursing home and 36 to home.<br>• By the end of one year, 37 of the 73 survivors will die. In other words, on average, only 36 out of 100 will survive one year after hospitalization.	**On average,**<br>• Very few people may survive without having to use a breathing tube, maybe 3 out of 100 people. In other words, 97 out of 100 people will not survive.

Survival
(after breathing machine)

In Total, by the end of the year

Of the 100 people who choose the breathing tube

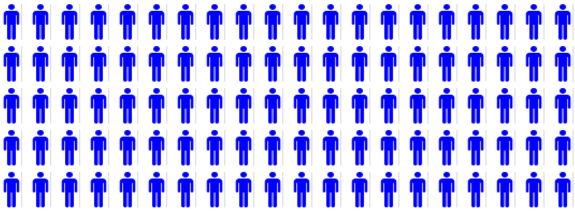

On average, 73 people will survive the first hospitalization

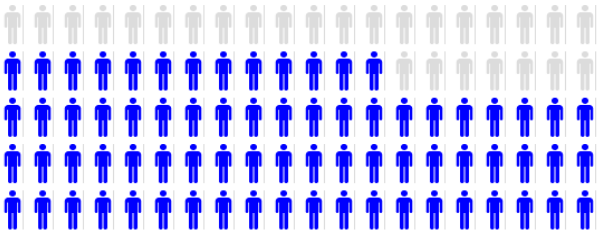

And, by the end of year 1, on average, 36 people will be alive.

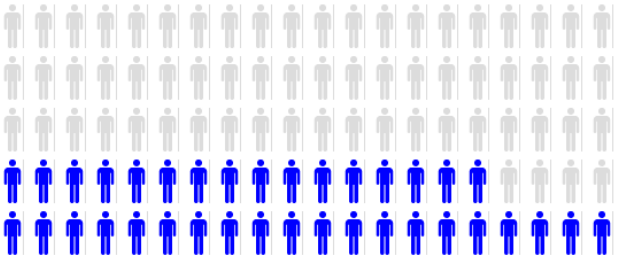

Quality of Life
(after breathing machine)

On Average, what will happen to those who survive right after receiving the breathing tube?

On average 73 people will survive (27 will not survive)
On average 37 of the survivors will go to a nursing home
On average 36 will go back home

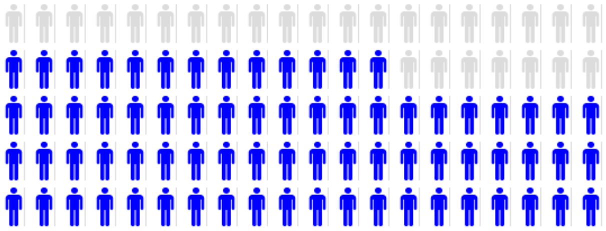

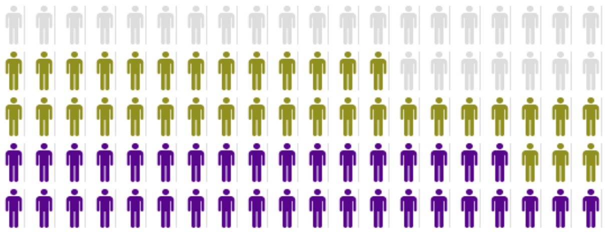

On average 23 of the survivors will be **hospitalized** 3 or more times within the next year

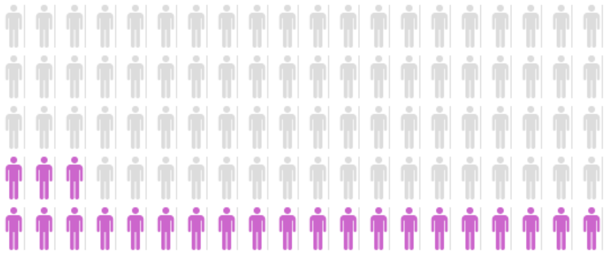

Supplementary Table 1: Additional Demographics

Table 1.1 Patient participants (n=38), n (%)		Results
**Access to home computer with internet**	Yes	30 (79.0%)
**Comfort with using the internet**	Not at all comfortable	15 (39.5%)
	Somewhat comfortable	5 (13.2 %)
	Mostly comfortable to extremely comfortable	18 (47.4%)
**Type of insurance**	Public insurance (Medicaid, Medicare, VA, etc.)	31 (81.6%)
	Private insurance (United, BlueCross, etc.)	16 (42.1%)
**Current living arrangement**	Live alone	11 (29.0%)
	Live with a spouse or partner/family member	24 (63.2%)
**Self-rating of health in general**	Excellent/very good	6 (15.8%)
	Good	9 (23.8%)
	Fair	16 (42.1%)
	Poor	7 (18.4%)
**Quality of Life - scale of 0-10**		mean 7.08 (s.d 2.9)
[illegible]	[illegible]	[illegible]
	Moderate	7 (18.9%)
	Severe	17 (46.0%)
	Very Severe	8 (24.3%)
**Number of hospital admissions in the past year**	0	16 (42.1%)
	1 or 2	13 (34.2%)
	3 or more	9 (23.7%)
**Comorbidities**	Hypertension/High blood pressure	28 (73.7%)
	Heart Disease	17 (46.0%)
	Cancer	7 (18.4%)
	Depression	13 (34.2%)
**Advance Care Planning**	Advance Directive	16 (42.1%)
	Spoken to doctor about Advance Directives	6 (15.8%)
	Healthcare proxy	27 (71.1%)
**Numeracy questions (answered correctly)**	Coin Toss	25 (65.8%)
	Marathon	25 (65.8%)
	School Raffle	23 (60.5%)
	100 People with Disease X<br>How many are women	30 (79.0%)
	**How many more men than women?**	15 (40.5%)
**Numeracy**	Low (≥ 3 wrong)	19 (50.0%)
**Cognitive Impairment (MMSE)**	No cognitive impairment (24-30 points)	30 (94.1%)

Table 1.2 Clinician Participants (n=11), n (%)		Results
**Age**		43.6 (s.d. 10.6)
**Gender**	Female	8 (72.7%)
**Years since residency completion**		17.6 (s.d. 11.0)
**Race/ethnicity**	White	5 (45.5%)
	Black/African American	1 (9.1%)
	Hispanic/Latino	2 (18.2%)
	Asian/Asian American	3 (27.3%)
**Patients with severe COPD with whom the clinician has discussed mechanical ventilation and Advance Directives**	Very few	2 (18.2%)
	Some	4 (36.4%)
	About Half	4 (36.4%)
	Most	1 (9.1%)

Table 1.3 Surrogate Participants (n=7)		Results
**Relationship to patient**	Spouse	4 (57.1)
	Parent	1 (14.3)
	Sibling	1 (14.3)
	Other	1 (14.3)

Supplementary Table 2 – Distress Experienced While Viewing Decision Aid

Distress Scenario	Sample Text from Clinician Patient Encounter	Significant patient characteristics
Patient expresses distress, and asks to stop viewing decision aid	**Clinician participant: If you feel that this is too stressful for you or you're getting too anxious, we can certainly stop. You tell me what you want to do.**<br>Patient participant: It's a lot to take. I feel I've had enough.	Patient: Severe COPD<br>Deceased by time of 1-month follow up
Patient expresses distress but continues after clinician uses languages acknowledging the difficulty of conversation	**Clinician participant: So in the next few slides, if you would like, we can talk about the upside and downside of breathing tubes, so you can make a decision that's right for you. Do you want to go on?**<br>Patient participant: I don't know if I want to go on.<br>**Clinician participant: No? Okay. You don't want to talk about it?**<br>Patient participant: No, because I don't feel comfortable yet about talking about this. Because I really wasn't expecting all this.<br>**Clinician participant: Yeah, of course, and I realize this conversation—the whole point of this, again, is because it's a sensitive topic, and realistically this is—a lot of people aren't informed. They don't know what the difference is. Yeah, it's not specifically for—**<br>Patient participant: No, no, I hear.<br>**Clinician participant: So I realize this conversation might be making you anxious or upset, but I want—before you go on, I want to let you know that's normal to feel anxiety or to be afraid of thinking—becoming very sick, so I don't mean to upset you, but I want to make some time to make sure that you and your family are prepared. The best choice is—**<br>Patient participant: This is stuff that we never discuss…..<br>**Clinician participant: Okay? Should we continue? It's up to you. I mean…**<br>Patient participant: Go ahead. Let's see.	Patient: severe COPD,<br>Never considered decision before
Patient expresses distress at thought of having her children seeing her intubated	Patient participant: I don't want to see my kids…they would suffer if they saw me going through something like that.<br>**Clinician participant: Going through—yeah, so that's why the topic itself is so sensitive.**<br>Patient participant: You know the people that are being left behind or what have you, the ones that you love, you don't—because I know how they felt when my husband was taking his last breath, and I had to make the decision for them to turn it off, but I held on as long as I could for their sake because, you know, it was a hard thing, but somebody has to make the decision…<br>**Clinician participant: So let's review what you learned so far, so in your own words, can you summarize what you learned so far about intubation and comfort measures? Like, for example, what would you say to your family about what you've seen today?**<br>Patient participant: You know, my husband was sick for many years, and I was back and forth to [the hospital] from 2003 back and forth until he died in 2012. I myself would not want to put my kids through going to the hospital every day, watching me slowly but surely just…I don't want—my body couldn't take it because I couldn't take watching them see it. I wouldn't want to stay here. I…I…I just don't wanna.	Patient: COPD severe<br>Experienced having to remove her husband from life support

Supplementary Table 3 – Patient Interest in Trial Intubation

Context for discussion of trial intubation	Sample Text from Clinician Patient Encounter
**Patient's evolution toward trial intubation from clinic visit to 1-month follow up interview**	During Clinic Visit:<br>**Clinician: Have you ever given any, I mean, as someone who has seen that in your own mother, and knows that—I mean, have you ever given any thought to if your COPD, if you got worse, if you would want to be put on a machine or—**<br>Patient: The way I rationalize that, like, no. No machines, you know.<br><br>Same patient at 1-month follow-up :<br>**Interviewer: Did you see the part where you have those things that you can kind of, scroll—it is called the "values clarification exercises" where it looks at what is most important to you and you can use the sliding scale?**<br>Patient: Oh yeah, I did that.<br>**Interviewer: Okay. Was that helpful?**<br>Patient: Very, yeah.<br>**Interviewer: Okay. So if you were to have a bad exacerbation, you would want to be treated with a breathing machine, at least, to try it?**<br>Patient: Uh, yeah… I think other questions that I had the…I'm trying to…I think the most important question for me was…about the breathing machine could I have an Advance Directive indicating timeframe that I want to be put on a breathing machine. For example, like, ten days…(or something?), like, two weeks, [unintelligible]. You can do that right?<br>**Interviewer: Like a trial with it?**<br>Patient: For example, my daughter would communicate my wishes if I need to go on the breathing machine, I would do so, but not more than one month [unintelligible], you know?<br>**Interviewer: Right, so you wouldn't want to be on it for a year, you would say, give it a month and then that is it?**<br>Patient: Right.<br>**Interviewer: So, did your decision change since you saw this information with the doctor?**<br>Patient: Absolutely, because I really hadn't thought about it in depth, you know.
**Patient bringing up trial intubation while viewing the decision aid with clinician**	Patient participant: "Is it a permanent thing, this breathing tube? Is there some kind of form or consent form, or something that shows like a proxy-type thing, that says, this is what I want unless you feel like I will be a vegetable, or I won't have any quality of life?"<br>**Clinician participant: Right. And part of the beauty of this new form is, it's, "We want this, we want this, and I want a trial of this. And if it looks like it's not working, then make me comfortable." It is that we don't necessarily have to pull the proverbial plug, we can kind of see how you do…"**
**Patient discussing trial intubation with researcher during 1-month follow up interview**	**Interviewer: If you were to have a bad COPD exacerbation would you want to be treated with the breathing machine?**<br>Patient participant: Yes.<br>**Interviewer: Okay. And has this decision changed since your conversation with your doctor at that visit? In other words, from seeing the decision aid?**<br>Patient participant: It's like I can be on the breathing machine, but if I feel that it's not good, or it hurts too much, it's not a good quality of life, or I can make—you know, I can make the decision to come off of it. So, you know, you're kind of open….<br>**Interviewer: Right.**<br>Patient participant: You know? So, it really is okay to start and see how it goes with that. I think that that's important to know. That you can start with it. If you feel that you don't want to do it, this is not how you want to live, you know, while you're on the breathing machine, you know, discuss it with a family member or somebody close to you and say, "listen I really…I can't do this." And more on a factual level than emotional level that it was…it's good that I have the choice. So, I'd definitely do it to start. And that was the decision I came to after meeting with you and Dr. [name].<br>**Interviewer: Okay, so yours is that you would—before that you really didn't have that decision in place?**<br>Patient participant: No. I wasn't sure.

Supplementary Table 4. Univariable Analysis: Associations between Communication and Outcomes

	SatCom1-10	SatCom11-20
**Change in knowledge**	-0.08 (p 0.66)	-0.14 (p 0.41)
**Change in motivation**	-0.08 (p 0.66)	-0.08 (p 0.65)
**DCS post**	-0.29 (p 0.09)	-0.46 (**p 0.005**)
**Change in DCS**	-0.01 (p 0.95)	0.11 (p 0.62)
**OPTION score**	0.23 (p 0.18)	0.28 (p 0.09)
Results represent Spearman's correlation coefficients and the corresponding p-values		

Supplementary Table 5 Associations between Outcomes and Patient Demographics

Outcomes	Age (34-60; 61-75; >75)	Marital Status (yes/no)	Sex	Ethnicity (Hispanic/ Black/ White)	Religion (yes/no)	Education level (5 levels)	Economic Class (5 levels)	English Fluent (yes/no)	Numeracy (high vs low)
**Change in knowledge**	5.4 (p 0.07)	4.7 (p 0.32)	0.6 (p 0.46)	1.3 (p 0.51)	0.1 (p 0.77)	9.1 **(p 0.05)**	4.3 (p 0.37)	0.2 (p 0.67)	2.0 (p 0.16)
**Change in motivation**	0.5 (p 0.79)	3.3 (p 0.50)	2.7 (p 0.1)	1.4 (p 0.49)	1.0 (p 0.31)	6.1 (p 0.19)	0.4 (p 0.98)	0.5 (p 0.5)	0.5 (p 0.56)
**Change in motivation at 1 month**	0.7 (p 0.71)	6.0 (p 0.19)	0.4 (p 0.52)	3.0 (p 0.22)	1.7 (p 0.19)	5.2 (p 0.27)	3.8 (p 0.43)	0.5 (p 0.48)	0.1 (p 0.75)
**DCS post**	2.0 (p 0.37)	6.4 (p 0.17)	0.0 (p 0.93)	3.7 (p 0.16)	2.4 (p 0.12)	8.1 (p 0.09)	3.9 (p 0.53)	1.9 (p 0.17)	2.1 (p 0.14)
**Change in DCS**	0.6 (p 0.74)	0.8(p 0.94)	0.0 (p 0.97)	3.8 (p 0.15)	3.9 **(p 0.05)**	5.3 (p 0.25)	1.8 (p 0.77)	1.3 (p 0.25)	0.4 (p 0.55)
Results from Nonparametric Kruskal-Wallis test with the corresponding p-values									

Supplementary Table 5 Univariable Analysis: Associations between Outcomes and Patient Self-Rated Health

**Outcomes**	**Self-rated health (5 levels)**	**Self-rated QOL (1-5 vs 6-10)**	**Self-rated COPD level**	**Hospitalizations in past yr (<=2 vs >2)**
**Change in knowledge**	6.42(p 0.17)	6.1 **(p 0.02)**	6.2 (p 0.19)	1.1 (p 0.30)
**Change in motivation**	3.4 (p 0.49)	0.0 (p 0.96)	5.0(p 0.29)	2.2 (p 0.14)
**Change in motivation at 1 month**	5.7 (p 0.22)	6.3 **(p 0.01)**	2.7(p 0.6)	5.1 (p 0.02)
**DCS post**	7.2 (p 0.13)	6.0 **(p 0.02)**	3.1 (p 0.55)	1.8 (p 0.18)
**Change in DCS**	0.4 (p 0.98)	1.8 (p 0.18)	1.5 (p 0.67)	0.3 (p 0.59)

Results from Nonparametric Kruskal-Wallis test with the corresponding p-values

Supplementary Figure 2: Screen shots of the InformedTogether Decision Aid

Pages showing general prognostic estimates and introducing the breathing tube

Northwell Health InformedTogether COPD Decision Aid | Patient | Resources | Language | ⇄ | Logout | Comments

Add a Note

Next | Page 1 / 14

General COPD estimates

First, **lets review some general estimates of outcomes** (based on clinical studies and NOT on mathematical modeling) for patients with severe COPD:

4 year survival
On average, patients with severe COPD have a **less than 60%** chance of being alive in 4 years time.

1 year hospitalization
On average, patients with severe COPD have a **greater than 50%** chance of being hospitalized for an acute COPD exacerbation within the next year.

6 month survival
On average, patients who are hospitalized and admitted to the ICU with respiratory failure due to COPD have a **62%** six month survival.

References:
Celli et al, NEJM 2004; 3 50:1005-12
Fitzgerald et al. Can Respir J 2007;14(3):145-152
Wildman et al. Thorax 2009; 64:128-32
Hajizadeh et al. Thorax 2014; doi:10.1136/thoraxjnl-2014-205248

Next

Northwell Health InformedTogether COPD Decision Aid | Patient | Resources | Language | ⇄ | Logout | Comments

Add a Note

Back | Next | Page 2 / 14

What does it mean to be on a breathing tube for life support?

In the hospital, patients may need to have a breathing tube which goes into their mouth and into their "windpipe". This tube is attached to a breathing machine. This is called intubation. The breathing machine is also called a mechanical ventilator.

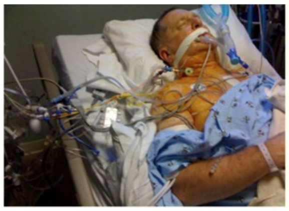

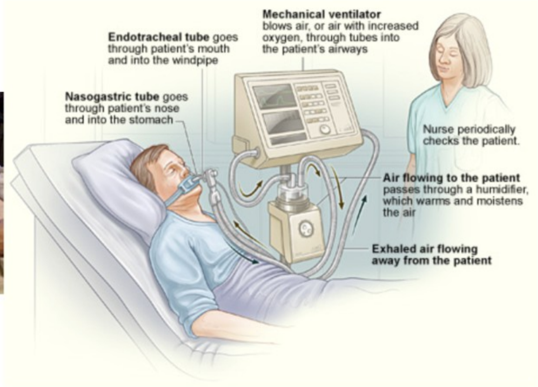

Back | Next

Page showing the choices to be made and the risks and benefits of each treatment option

Northwell Health | InformedTogether COPD Decision Aid | Patient | Resources | Language | Logout | Comments

Add a Note

Back | Next | Page 3 / 14

Choices if you can no longer breathe on your own*

- If you can no longer breathe on your own, despite other treatments, you can choose either (a) to be connected to a breathing machine, or (b) to accept comfort care only (no machine).

Breathing Machine	Comfort Care Only
If you choose to be connected to a breathing machine, a tube will be inserted into your windpipe, and the other end of the tube would be attached to a machine that breathes for you by blowing air into your lungs and sucking it out.	If you choose not to be connected to a breathing machine, you will continue to receive medical care, as before, plus additional medications, as needed, to make you comfortable. It is very important for you to understand that you would receive medications that prevent a feeling of suffocation even when you are not able to take in enough oxygen.

- **A patient who chooses not to get a breathing tube may not survive.**
- **So, why would anyone make this choice?** The reason is that a breathing tube can be uncomfortable and cause problems (complications). There are risks that we will talk about in the next slide

Back | Next

Northwell Health | InformedTogether COPD Decision Aid | Patient | Resources | Language | Logout | Comments

Add a Note

Back | Next | Page 4 / 14

Breathing Tube risks compared to Comfort Measures Only risks *

Breathing Tube Risks	Confort Measures Only Risks
Some patients will not survive despite the breathing tube. We will review the percent of people who most likely won't survive in a few slides.	Most patients who are treated with comfort measures only instead of a breathing tube **will not survive**, but the medications will support a peaceful death.
20-30-% of patients cannot come off the breathing machine and **will not be able to return back home.** At this point the breathing tube is usually moved to the neck (a **tracheostomy).** We will review this more in the next two slides.	
100% of patients **cannot talk** while on the breathing machine (although they can point to words and can write)	
100% of patients **cannot eat** while on breathing machine (although they get nutrition through a feeding tube)	
100% **cannot walk** for most of the time they are on the breathing machine	
Some patients have **discomfort** with the breathing tube being in their mouth.	

Pages setting up the dicussion of prognostic estimates, stating what would happen “on average” to those people who choose to get a breathign tube.

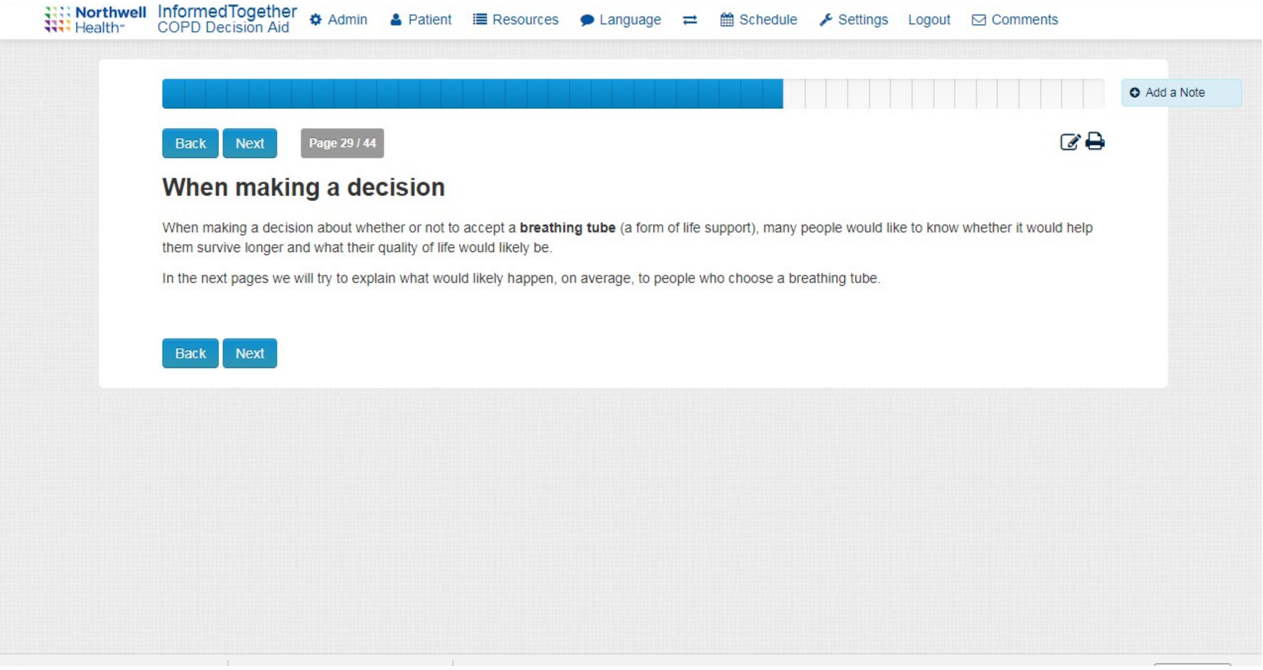

Pages setting up the dicussion of prognostic estimates

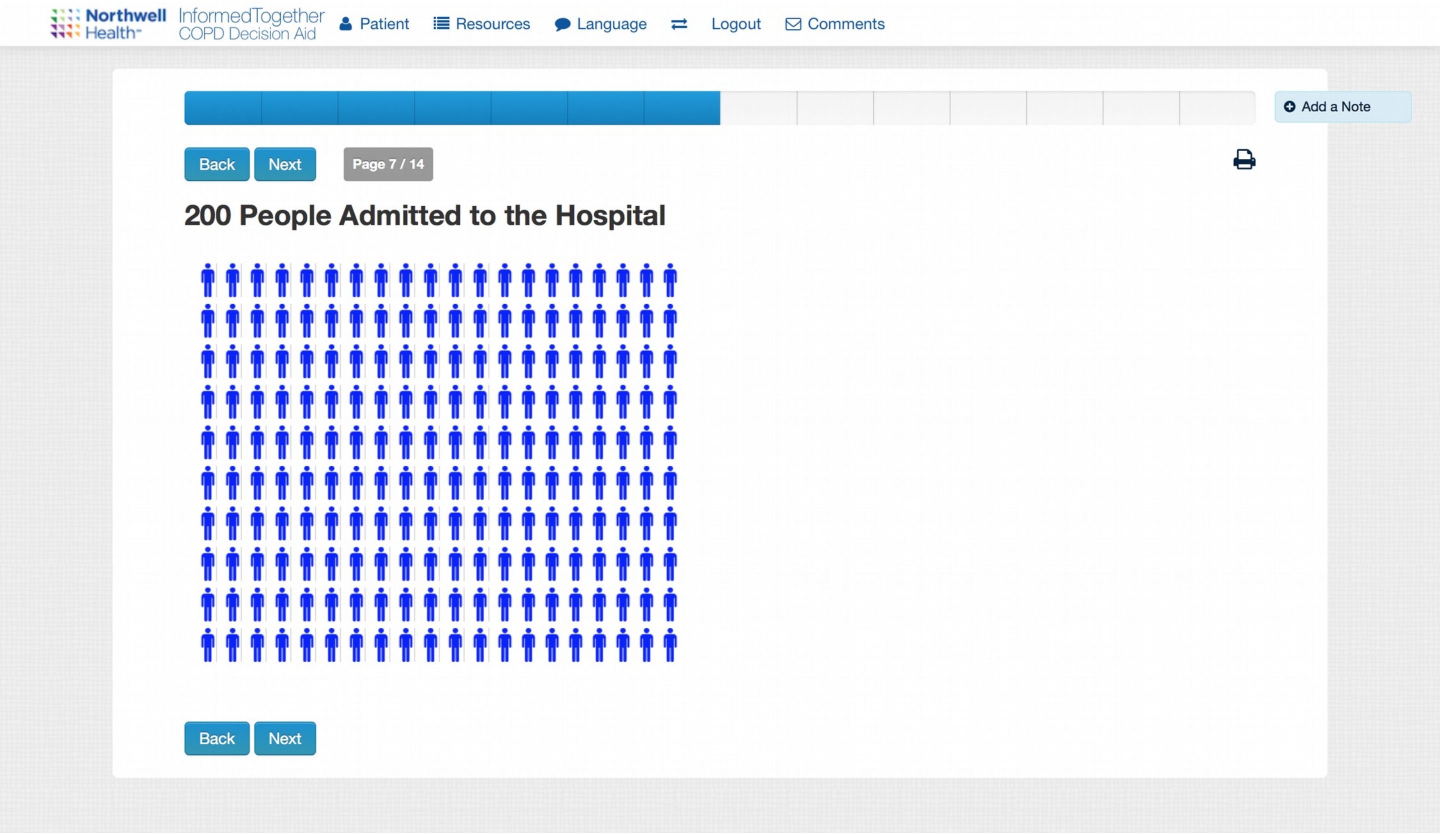

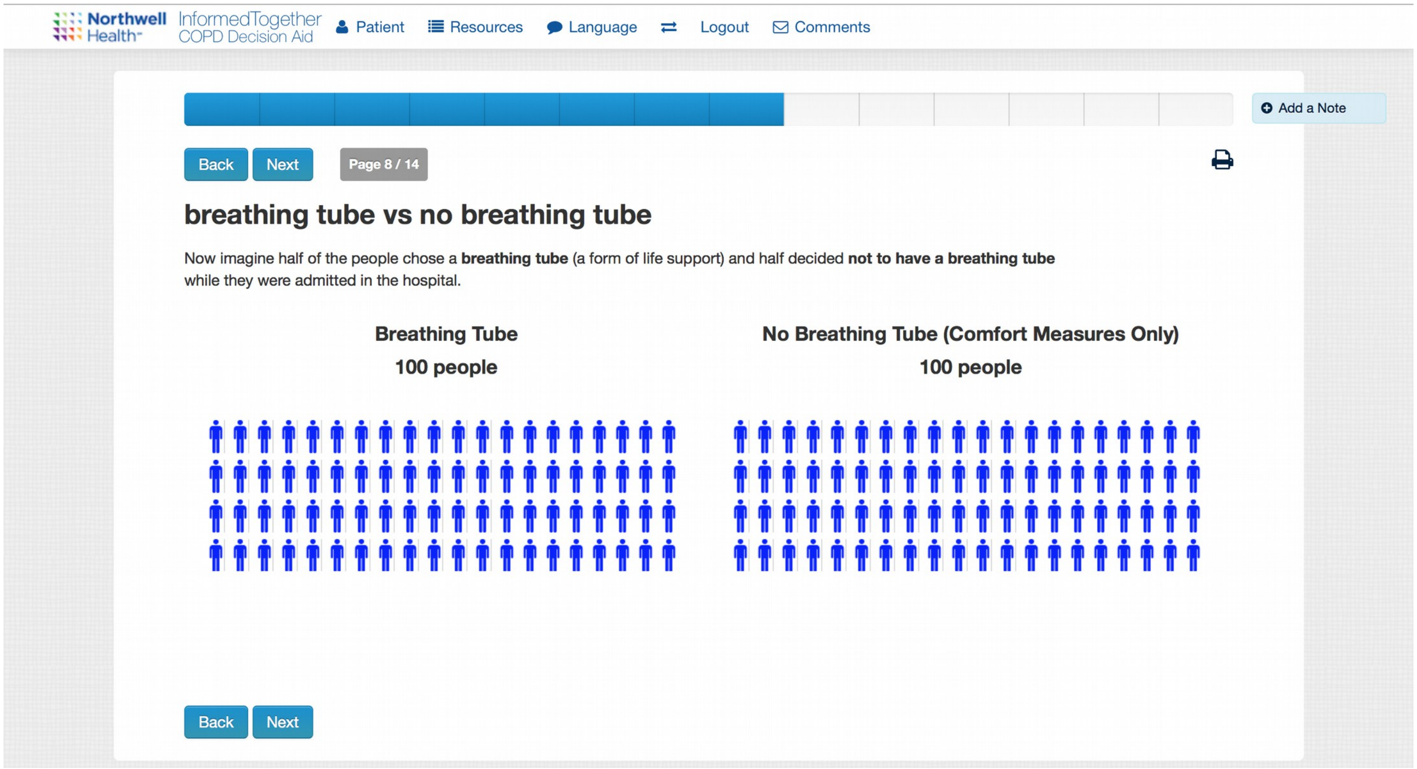

Add a Note

Back Next Page 9 / 14

On average, how many will survive right after they are admitted to the hospital?

Breathing Tube (for a person of your age)

On average, 68 people will survive (32 will not survive)

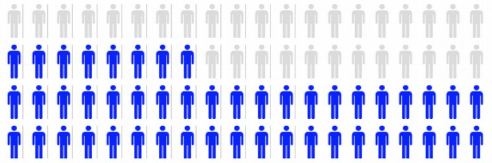

No Breathing Tube (Comfort Measures Only)

On average very few people will survive (Almost 100 people will not survive)

Back Next

On average, what will happen to those who survive right after receiving the breathing tube?

On average, 68 people will survive (32 will not survive)

On average, 34 of the survivors will go to a nursing home

On average, 34 will go back home

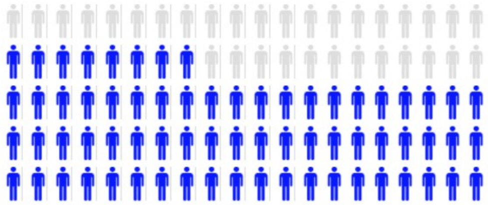

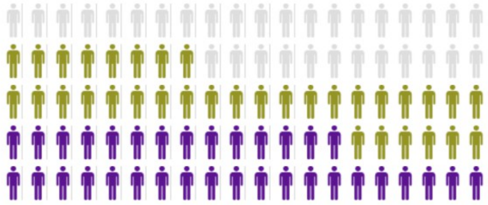

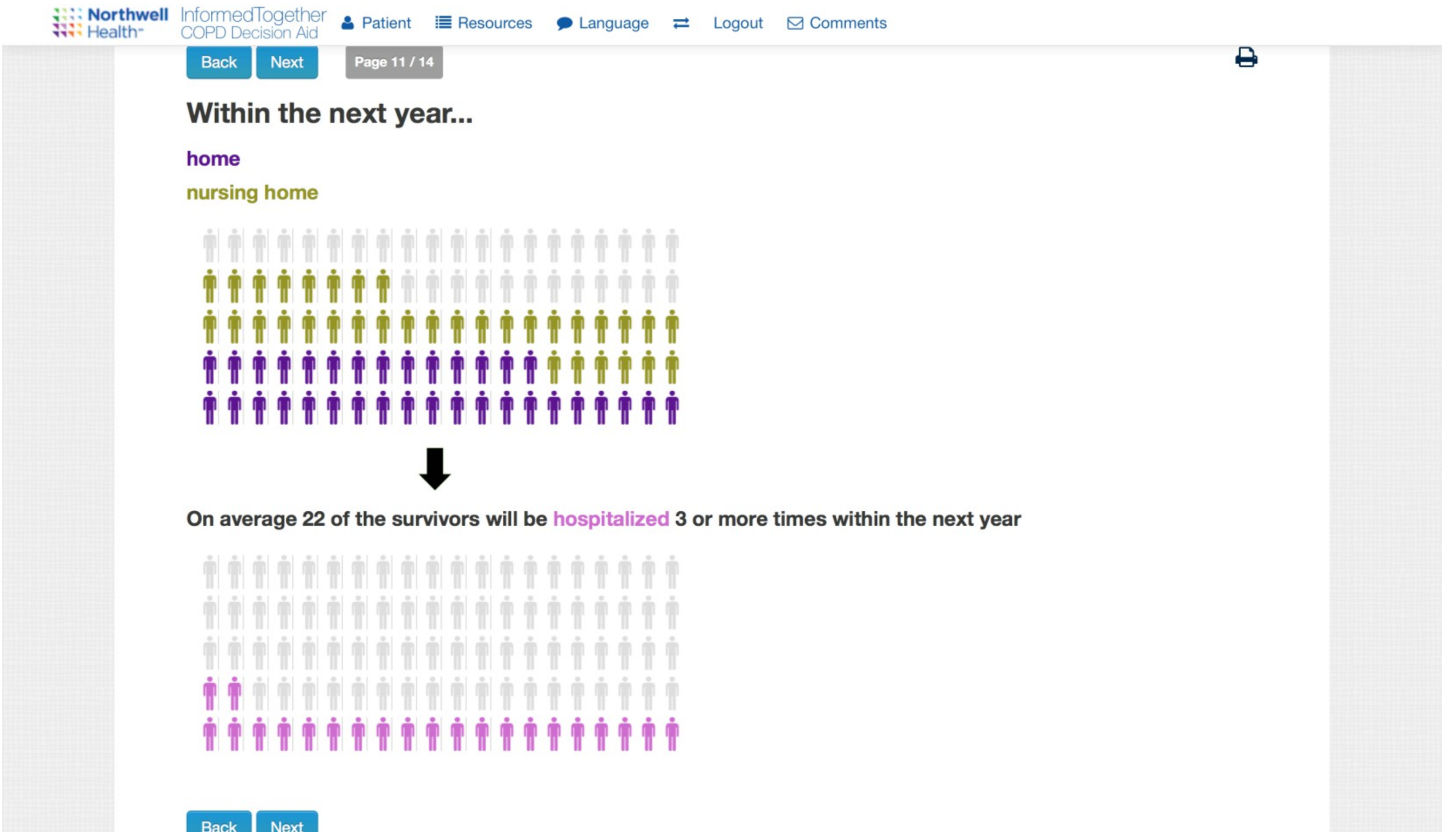

Page with preference elicitation excercise

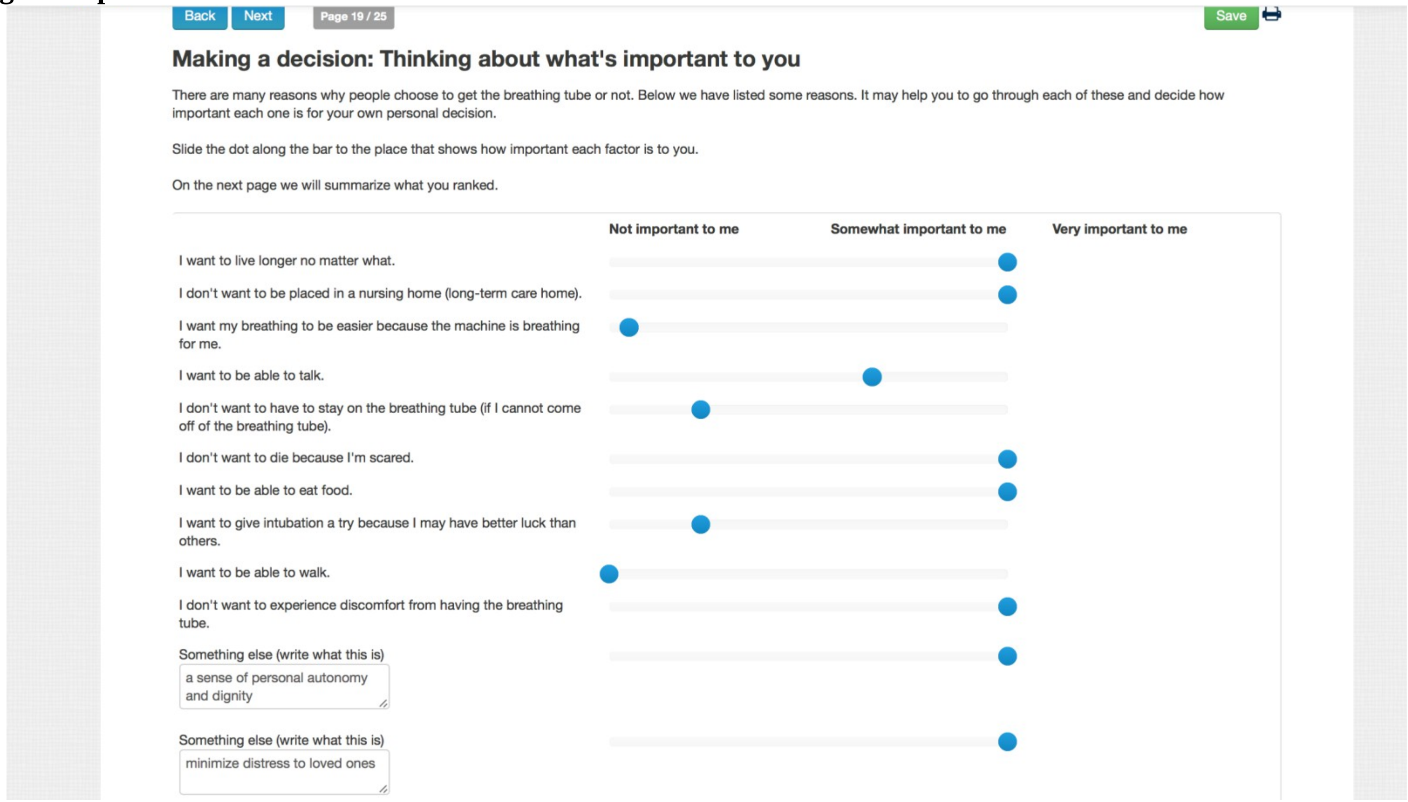

Page explaining how prognostic estimates were dervied

Northwell Health InformedTogether COPD Decision Aid | Admin | Patient | Resources | Language | Schedule | Settings | Logout | Comments

Back | Page 44 / 44

Information sources that were used to calculate estimates

The InformedTogether decision aid was developed using the best available research evidence. The estimates used in the decision aid are from a mathematical model that projects outcomes for COPD patients.
In simple terms, we looked at all the studies of patients with COPD who received a breathing tube for life support and put together all the outcomes in all of these studies. This means we looked at how many people survived and how long they lived as well as how many people had to go to a nursing home. Separately we tried to make sure that these numbers were as accurate as they could be by looking at what happened to all Medicare patients with severe COPD who received a breathing tube for life support.

A list of our information sources is below.

- Hajizadeh N, Crothers K, R Scott Braithwaite (2013). Using modeling to inform patient-centered care choices at the end of life. *Journal of Comparative Effectiveness Research*. 2(5):497-508. Article link.
- Hajizadeh N, Crothers K, R Scott Braithwaite (2012). Informing Shared Decisions about Advance Directives for Patients with Severe Chronic Obstructive Pulmonary Disease: A Modeling Approach. *Value in Health*. 15(2):357-66. Article link.
- Hajizadeh N, Crothers K, R Scott Braithwaite (2010). A theoretical decision model to help inform advance directive discussions for patients with COPD. *BMC Med Inform Decis Mak*. 10:75. Article link.
- Wildman M, Sanderson C, Groves J, et al. (2009). Survival and quality of life for patients with COPD or asthma admitted to intensive care in a UK multicentre cohort: the COPD and Asthma Outcome Study (CAOS). *Thorax*. 64:128-32. Article link.
- FitzGerald F, Haddon J, Bradly-Kennedy C, et al. (2007). Resource Use Study in COPD (RUSIC): A prospective study to quantify the effects of COPD exacerbations on health care resource use among COPD patients. *Can Respir J*. 14(3):145-152. Article link.
- Celli B, Cote C, Marin J, et al. (2004). The Body-Mass Index, Airflow Obstruction, Dyspnea, and Exercise Capacity Index in Chronic Obstructive Pulmonary Disease. *New Engl J Med*. 350:1005-12. Article link.

The mathematical model was tested to see if the outcomes it predicted were similar to a Medicare patient group's outcomes (validation). Details can be found in the article below.

- Hajizadeh N, Goldfeld K, Crothers K. (2014) What Happens To Advanced Stage COPD Patients Who Get Intubated For COPD Exacerbation? A One-Year Retrospective Follow up Study. *Thorax*. Article link.

Supplementary Figure 3a: Feasibility and Acceptability Questionnaires for Patients

Feasibility and Acceptability

I am now going to ask you some questions about the use of the decision aid in your clinic visit today.

FA1. Using the decision aid got in the way of the care I received in this clinic

a. __ Strongly agree
b. __ Somewhat agree
c. __ Not sure/neutral
d. __ Somewhat disagree
e. __ Strongly disagree

FA2. Please explain your answer.

FA3. My doctor and I were able to use the decision aid together during the clinic visit.

a. __ Strongly agree
b. __ Somewhat agree
c. __ Not sure/neutral
d. __ Somewhat disagree
e. __ Strongly disagree

FA4. How helpful is the decision aid?

a.__ Very helpful
b.__ Somewhat helpful
c.__ A little helpful
d.__ Not helpful

e. What was it helpful for?
____

FA5. How helpful was the decision aid for giving you information you need to make a decision about whether to accept a breathing tube.

a.__ Very helpful
b.__ Somewhat helpful
c.__ A little helpful
a. __ Not helpful

FA6. Would you recommend the decision aid to others?

a.__ Definitely
b.__ Probably
c.__ Probably not
d.__ Definitely not

b. Why or why not?

FA7. How clear was the information in the decision aid?

a. ___ Everything was clear
b. ___ Most things were clear
c. ___ Some things were clear
d. ___ Many things were unclear

FA8. I was satisfied with the discussion I had with my doctor using the InformedTogether decision aid.
a. __ Strongly agree
b. __ Somewhat agree
c. __ Not sure/neutral
d. __ Somewhat disagree
e. __ Strongly disagree

FA9. I would like my doctor to use the InformedTogether decision aid with me again at a future clinic visit.
a. __ Strongly agree
b. __ Somewhat agree
c. __ Not sure/neutral
d. __ Somewhat disagree
e. __ Strongly disagree

FA10. It's appropriate for doctors to use the InformedTogether decision aid with patients like me (with severe COPD).
a. __ Strongly agree
b. __ Somewhat agree
c. __ Not sure/neutral
d. __ Somewhat disagree
e. __ Strongly disagree

FA11. I am planning to use the decision access the decision aid online at home.
a. __ Strongly agree
b. __ Somewhat agree
c. __ Not sure/neutral
d. __ Somewhat disagree
e. __ Strongly disagree

FA12. I am planning to talk to a family member about the information my doctor showed me in the decision aid.
a. __ Strongly agree
b. __ Somewhat agree
c. __ Not sure/neutral
d. __ Somewhat disagree
e. __ Strongly disagree

FA13. I am planning to show my family member the decision aid on the computer.
a.__ Strongly agree
b.__ Somewhat agree
c.__ Not sure/neutral
d.__ Somewhat disagree
e.__ Strongly disagree

FA14. Other COPD patients would like to have their doctor use the InformedTogether decision aid during their clinic visit.

a.__ Strongly agree
b.__ Somewhat agree
c.__ Not sure/neutral
d.__ Somewhat disagree
e.__ Strongly disagree

Supplementary Figure 3b: Feasibility and Acceptability Questionnaires for Clinicians

Feasibility and Acceptability

I am now going to ask you a few questions about the feasibility of using this decision aid with your patients during their clinic visits.

FA1. How helpful is the decision aid?
__ Very helpful
__ Somewhat helpful
__ A little helpful
__ Not helpful

What was it helpful for?

FA2. How helpful was the decision aid for giving you information you need to make a decision about whether to accept a breathing tube.
__ Very helpful
__ Somewhat helpful
__ A little helpful
__ Not helpful

FA3. Would you recommend the decision aid to others?
__ Definitely
__Probably
__Probably not
__Definitely not

Why or why not?

FA4. How clear was the information in the decision aid?
___ Everything was clear
___ Most things were clear
___ Some things were clear
___ Many things were unclear

FA5. I was satisfied with the discussion I had with my patient using the InformedTogether decision aid.
__ Strongly agree __ Somewhat agree __ Not sure/neutral __ Somewhat disagree __ Strongly disagree

FA6. I plan to use the InformedTogether decision aid with this patient again at a future clinic visit.
__ Strongly agree __ Somewhat agree __ Not sure/neutral __ Somewhat disagree __ Strongly disagree

FA7. It's appropriate for doctors to use the InformedTogether decision aid with their severe COPD patients.
__ Strongly agree __ Somewhat agree __ Not sure/neutral __ Somewhat disagree __ Strongly disagree

FA8. Use of the decision aid negatively affects the flow of care I provide to my patients.
__ Strongly agree __ Somewhat agree __ Not sure/neutral __ Somewhat disagree __ Strongly disagree

FA9. I am able to easily integrate use of the InformedTogether decision aid into regular clinic visits with my patients.
__ Strongly agree __ Somewhat agree __ Not sure/neutral __ Somewhat disagree __ Strongly disagree

FA10. Using the decision aid allows me to provide better care for my patients with severe COPD.
__ Strongly agree __ Somewhat agree __ Not sure/neutral __ Somewhat disagree __ Strongly disagree

FA11. Using the decision aid improves my communication about their preferences for life sustaining treatments with my patients with severe COPD.
__ Strongly agree __ Somewhat agree __ Not sure/neutral __ Somewhat disagree __ Strongly disagree

FA12. I am able to use the decision aid as part of my clinical practice.
__ Strongly agree __ Somewhat agree __ Not sure/neutral __ Somewhat disagree __ Strongly disagree

FA13. Using the InformedTogether decision aid with severe COPD patients is consistent with our organizational culture.
__ Strongly agree __ Somewhat agree __ Not sure/neutral __ Somewhat disagree __ Strongly disagree

FA14. Using the InformedTogether decision aid improves the patient care experience at this pulmonary clinic.
__ Strongly agree __ Somewhat agree __ Not sure/neutral __ Somewhat disagree __ Strongly disagree

FA15. I am enthusiastic about using the InformedTogether decision aid with other patients.
__ Strongly agree __ Somewhat agree __ Not sure/neutral __ Somewhat disagree __ Strongly disagree

FA16. My patients with severe COPD at this clinic would like to have me use the InformedTogether decision aid with them during their clinic visit.
__ Strongly agree __ Somewhat agree __ Not sure/neutral __ Somewhat disagree __ Strongly disagree

FA17. Other doctors in this clinic who treat COPD patients like using the InformedTogether decision aid with their COPD patients.
__ Strongly agree __ Somewhat agree __ Not sure/neutral __ Somewhat disagree __ Strongly disagree

FA18. Please indicate what percentage of the decision aid you went through with your patient?
__ 0-25% __ 25-50% __ 50-75% __ 75-100%

FA19. Did you encounter any technical difficulties while using the decision aid?
__ Yes __ No

If yes, please describe briefly ____
____

FA20. Did you experience any problems while using the decision aid (other than technical difficulties described above)?
__ Yes __ No

If yes, please describe briefly ____
____

FA21. Do you have any other feedback about using the InformedTogether decision aid?

____
____
____
