# Supplementary material for: A Decision Aid to Support Shared Decision Making About Mechanical Ventilation in Severe Chronic Obstructive Pulmonary Disease Patients (InformedTogether): Feasibility Study
Source: J Particip Med. 2018 May 14;10(2):e7. doi: 10.2196/jopm.9877 (PMC7251980; doi:10.2196/jopm.9877)

MA2: InformedTogether Option Grid for a COPD Patient Age Range 66-70 Years Old

Here are some answers to questions you may have when choosing between a Breathing Machine versus Comfort Measures Only (palliative care) when you have a severe COPD exacerbation and can't breathe.

Frequently asked questions	**Breathing Machine After A Severe Exacerbation**	**No Breathing Machine After A Severe Exacerbation (Comfort Care Only)**
**Tell me more about each of my options.**	A breathing tube goes into a patient's mouth and into their windpipe. This tube is attached to a breathing machine which blows air into the airway. This is called **intubation/mechanical ventilation and** is one kind of life support.	Comfort Care Only means a patient has chosen NOT to have any life support machines. In this case patients are treated with pain medications and other treatments to make them as comfortable as possible. This is also called palliative/hospice care.
**When are these treatment options offered to me?**	When you are not able to breathe and other treatments in the hospital (such as oxygen, inhalers, steroids and mask ventilation/bipap) do not work.	
**Will it help me to live longer?**	A breathing machine cannot cure COPD, but it may enable you to live longer by helping you breathe when you cannot do so on your own.	Most people who choose NOT to have any life support will not survive, but instead will have the care focused on a peaceful death.
**Will it be painful?**	A breathing machine can cause problems and make you uncomfortable. Doctors will give you pain medications and medications to help with this discomfort.	With Comfort Care, all treatments are focused on pain relief and medicines are given so you don't feel like you are having a hard time breathing.
**What are the benefits?**	• You may live longer.<br>• Breathing is usually easier because a machine will be doing the breathing for you.	• You will be given medication to treat pain, trouble breathing and help you to feel relaxed.<br>• The medications will support a peaceful death
**What are the risks?**	Although patients may live longer**,**<br>• 20-30% of patients may not be able to come off the breathing machine and **will not be able to return back home**.<br>• 100% of patients **cannot talk** while on the breathing machine.<br>• 100% of patients **cannot eat** while on the breathing machine.<br>• **Almost 100%** cannot walk because they are attached to the breathing machine.	• Most patients who choose <u>not</u> to be treated with a breathing machine and choose comfort measures only, **will not survive**.
**What are the <u>possible</u> results?**	**On average,**<br>• About 73 out of 100 people will survive in the hospital.<br>• About 37 of the 73 survivors will be discharged to a nursing home and 36 to home.<br>• By the end of one year, 37 of the 73 survivors will die. In other words, on average, only 36 out of 100 will survive one year after hospitalization.	**On average,**<br>• Very few people may survive without having to use a breathing tube, maybe 3 out of 100 people. In other words, 97 out of 100 people will not survive.

Survival (after breathing machine)	Quality of Life (after breathing machine)

In Total, by the end of the year

Of the 100 people who choose the breathing tube

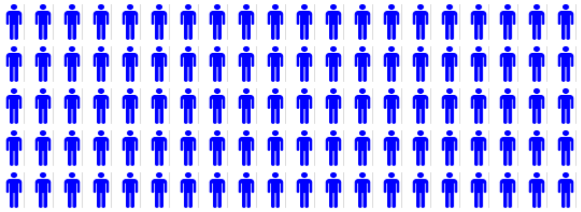

On average, 73 people will survive the first hospitalization

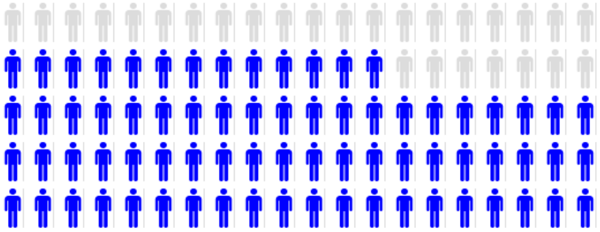

And, by the end of year 1, on average, 36 people will be alive.

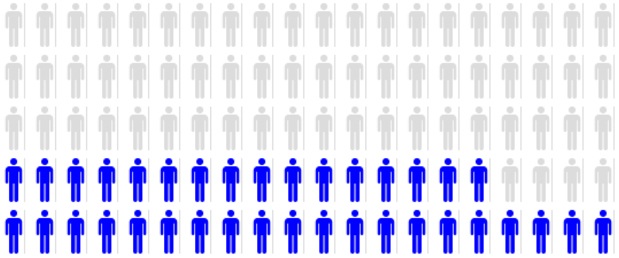

On Average, what will happen to those who survive right after receiving the breathing tube?

On average 73 people will survive (27 will not survive)
On average 37 of the survivors will go to a nursing home
On average 36 will go back home

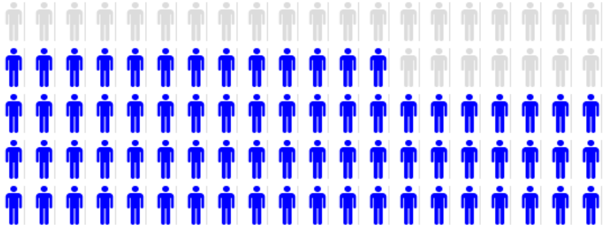

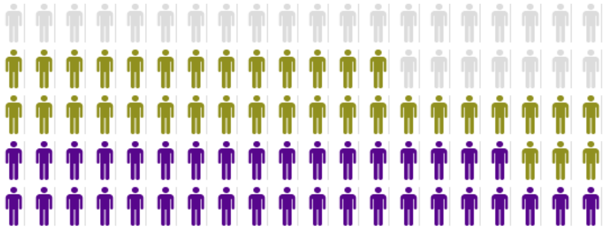

Within the next year…

On average 23 of the survivors will be **hospitalized** 3 or more times within the next year

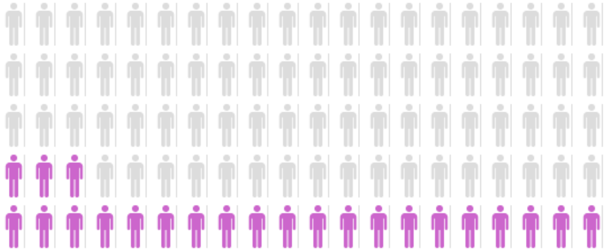

Supplement: Multimedia Appendix 2 [file jopm_v10i2e7_app2.pdf]
