# Supplementary material for: A Decision Aid to Support Shared Decision Making About Mechanical Ventilation in Severe Chronic Obstructive Pulmonary Disease Patients (InformedTogether): Feasibility Study
Source: J Particip Med. 2018 May 14;10(2):e7. doi: 10.2196/jopm.9877 (PMC7251980; doi:10.2196/jopm.9877)
Supplement: Multimedia Appendix 3 [file jopm_v10i2e7_app3.pdf]

MA3a Feasibility and Acceptability Questionnaires for Patients

Feasibility and Acceptability

I am now going to ask you some questions about the use of the decision aid in your clinic visit today.

FA1. Using the decision aid got in the way of the care I received in this clinic

a. __ Strongly agree
b. __ Somewhat agree
c. __ Not sure/neutral
d. __ Somewhat disagree
e. __ Strongly disagree

FA2. Please explain your answer.

FA3. My doctor and I were able to use the decision aid together during the clinic visit.

a. __ Strongly agree
b. __ Somewhat agree
c. __ Not sure/neutral
d. __ Somewhat disagree
e. __ Strongly disagree

FA4. How helpful is the decision aid?

a.__ Very helpful
b.__ Somewhat helpful
c.__ A little helpful
d.__ Not helpful

e. What was it helpful for?

____

FA5. How helpful was the decision aid for giving you information you need to make a decision about whether to accept a breathing tube.

a.__ Very helpful
b.__ Somewhat helpful
c.__ A little helpful
a. __ Not helpful

FA6. Would you recommend the decision aid to others?

a.__ Definitely
b.__ Probably
c.__ Probably not
d.__ Definitely not

b. Why or why not?

FA7. How clear was the information in the decision aid?
a. ___ Everything was clear
b. ___ Most things were clear
c. ___ Some things were clear
d. ___ Many things were unclear

FA8. I was satisfied with the discussion I had with my doctor using the InformedTogether decision aid.
a. __ Strongly agree
b. __ Somewhat agree
c. __ Not sure/neutral
d. __ Somewhat disagree
e. __ Strongly disagree

FA9. I would like my doctor to use the InformedTogether decision aid with me again at a future clinic visit.
a. __ Strongly agree
b. __ Somewhat agree
c. __ Not sure/neutral
d. __ Somewhat disagree
e. __ Strongly disagree

FA10. It's appropriate for doctors to use the InformedTogether decision aid with patients like me (with severe COPD).
a. __ Strongly agree
b. __ Somewhat agree
c. __ Not sure/neutral
d. __ Somewhat disagree
e. __ Strongly disagree

FA11. I am planning to use the decision access the decision aid online at home.
a. __ Strongly agree
b. __ Somewhat agree
c. __ Not sure/neutral
d. __ Somewhat disagree
e. __ Strongly disagree

FA12. I am planning to talk to a family member about the information my doctor showed me in the decision aid.
a. __ Strongly agree
b. __ Somewhat agree
c. __ Not sure/neutral
d. __ Somewhat disagree

e. __ Strongly disagree

FA13. I am planning to show my family member the decision aid on the computer.
a.__ Strongly agree
b.__ Somewhat agree
c.__ Not sure/neutral
d.__ Somewhat disagree
e.__ Strongly disagree

FA14. Other COPD patients would like to have their doctor use the InformedTogether decision aid during their clinic visit.
a.__ Strongly agree
b.__ Somewhat agree
c.__ Not sure/neutral
d.__ Somewhat disagree
e.__ Strongly disagree

MA3b: Feasibility and Acceptability Questionnaires for Clinicians

Feasibility and Acceptability

I am now going to ask you a few questions about the feasibility of using this decision aid with your patients during their clinic visits.

FA1. How helpful is the decision aid?

__ Very helpful
__ Somewhat helpful
__ A little helpful
__ Not helpful

What was it helpful for?

FA2. How helpful was the decision aid for giving you information you need to make a decision about whether to accept a breathing tube.

__ Very helpful
__ Somewhat helpful
__ A little helpful
__ Not helpful

FA3. Would you recommend the decision aid to others?

__ Definitely
__Probably
__Probably not
__Definitely not

Why or why not?

FA4. How clear was the information in the decision aid?

___ Everything was clear
___ Most things were clear
___ Some things were clear
___ Many things were unclear

FA5. I was satisfied with the discussion I had with my patient using the InformedTogether decision aid.
__ Strongly agree __ Somewhat agree __ Not sure/neutral __ Somewhat disagree __ Strongly disagree

FA6. I plan to use the InformedTogether decision aid with this patient again at a future clinic visit.
__ Strongly agree __ Somewhat agree __ Not sure/neutral __ Somewhat disagree __ Strongly disagree

FA7. It's appropriate for doctors to use the InformedTogether decision aid with their severe COPD patients.
__ Strongly agree __ Somewhat agree __ Not sure/neutral __ Somewhat disagree __ Strongly disagree

FA8. Use of the decision aid negatively affects the flow of care I provide to my patients.
__ Strongly agree __ Somewhat agree __ Not sure/neutral __ Somewhat disagree __ Strongly disagree

FA9. I am able to easily integrate use of the InformedTogether decision aid into regular clinic visits with my patients.
__ Strongly agree __ Somewhat agree __ Not sure/neutral __ Somewhat disagree __ Strongly disagree

FA10. Using the decision aid allows me to provide better care for my patients with severe COPD.
__ Strongly agree __ Somewhat agree __ Not sure/neutral __ Somewhat disagree __ Strongly disagree

FA11. Using the decision aid improves my communication about their preferences for life sustaining treatments with my patients with severe COPD.
__ Strongly agree __ Somewhat agree __ Not sure/neutral __ Somewhat disagree __ Strongly disagree

FA12. I am able to use the decision aid as part of my clinical practice.
__ Strongly agree __ Somewhat agree __ Not sure/neutral __ Somewhat disagree __ Strongly disagree

FA13. Using the InformedTogether decision aid with severe COPD patients is consistent with our organizational culture.
__ Strongly agree __ Somewhat agree __ Not sure/neutral __ Somewhat disagree __ Strongly disagree

FA14. Using the InformedTogether decision aid improves the patient care experience at this pulmonary clinic.
__ Strongly agree __ Somewhat agree __ Not sure/neutral __ Somewhat disagree __ Strongly disagree

FA15. I am enthusiastic about using the InformedTogether decision aid with other patients.
__ Strongly agree __ Somewhat agree __ Not sure/neutral __ Somewhat disagree __ Strongly disagree

FA16. My patients with severe COPD at this clinic would like to have me use the InformedTogether decision aid with them during their clinic visit.
__ Strongly agree __ Somewhat agree __ Not sure/neutral __ Somewhat disagree __ Strongly disagree

FA17. Other doctors in this clinic who treat COPD patients like using the InformedTogether decision aid with their COPD patients.
__ Strongly agree __ Somewhat agree __ Not sure/neutral __ Somewhat disagree __ Strongly disagree

FA18. Please indicate what percentage of the decision aid you went through with your patient?
__ 0-25% __ 25-50% __ 50-75% __ 75-100%

FA19. Did you encounter any technical difficulties while using the decision aid?
__ Yes __ No

If yes, please describe briefly ____
____

FA20. Did you experience any problems while using the decision aid (other than technical difficulties described above)?
__ Yes __ No

If yes, please describe briefly ____
____

FA21. Do you have any other feedback about using the InformedTogether decision aid?

____

____

____
