# Supplementary material for: A Decision Aid to Support Shared Decision Making About Mechanical Ventilation in Severe Chronic Obstructive Pulmonary Disease Patients (InformedTogether): Feasibility Study
Source: J Particip Med. 2018 May 14;10(2):e7. doi: 10.2196/jopm.9877 (PMC7251980; doi:10.2196/jopm.9877)
Supplement: Multimedia Appendix 4 [file jopm_v10i2e7_app4.pdf]

MA4: Additional Demographic Characteristics

Table 1.1 Patient participants (n=38), n (%)		Results
**Interpreter used when seeing the doctor**	No	35 (92.1%)
**Access to home computer with internet**	Yes	30 (79.0%)
**Comfort with using the internet**	Not at all comfortable	15 (39.5%)
	Somewhat comfortable	5 (13.2 %)
	Mostly comfortable to extremely comfortable	18 (47.4%)
**Type of insurance**	Public insurance (Medicaid, Medicare, VA, etc.)	31 (81.6%)
	Private insurance (United, BlueCross, etc.)	16 (42.1%)
**Current living arrangement**	Live alone	11 (29.0%)
	Live with a spouse or partner/family member	24 (63.2%)
**Self-rating of health in general**	Excellent/very good	6 (15.8%)
	Good	9 (23.8%)
	Fair	16 (42.1%)
	Poor	7 (18.4%)
**Quality of Life - scale of 0-10**		mean 7.08 (s.d 2.9)
**Self-rate severity of COPD**	Very mild/mild	4 (10.8%)
	Moderate	7 (18.9%)
	Severe	17 (46.0%)
	Very Severe	8 (24.3%)
**Number of hospital admissions in the past year**	0	16 (42.1%)
	1 or 2	13 (34.2%)
	3 or more	9 (23.7%)
**Comorbidities**	Hypertension/High blood pressure	28 (73.7%)
	Heart Disease	17 (46.0%)
	Cancer	7 (18.4%)
	Depression	13 (34.2%)
**Advance Care Planning**	Advance Directive	16 (42.1%)
	Spoken to doctor about Advance Directives	6 (15.8%)
	Healthcare proxy	27 (71.1%)
**Numeracy questions (answered correctly)**	Coin Toss	25 (65.8%)
	Marathon	25 (65.8%)
	School Raffle	23 (60.5%)
	100 People with Disease X<br>How many are women	30 (79.0%)
	**How many more men than women?**	15 (40.5%)
**Numeracy**	Low (≥ 3 wrong)	19 (50.0%)

**Cognitive Impairment (MMSE)**	No cognitive impairment (24-30 points)	30 (94.1%)

**Table 1.2 Clinician Participants (n=11), n (%)**		**Results**
**Age**		43.6 (s.d. 10.6)
**Gender**	Female	8 (72.7%)
**Years since residency completion**		17.6 (s.d. 11.0)
**Race/ethnicity**	White	5 (45.5%)
	Black/African American	1 (9.1%)
	Hispanic/Latino	2 (18.2%)
	Asian/Asian American	3 (27.3%)
**Patients with severe COPD with whom the clinician has discussed mechanical ventilation and Advance Directives**	Very few	2 (18.2%)
	Some	4 (36.4%)
	About Half	4 (36.4%)
	Most	1 (9.1%)
**Table 1.3 Surrogate Participants (n=7)**		**Results**
**Relationship to patient**	Spouse	4 (57.1)
	Parent	1 (14.3)
	Sibling	1 (14.3)
	Other	1 (14.3)
