# Supplementary material for: A Decision Aid to Support Shared Decision Making About Mechanical Ventilation in Severe Chronic Obstructive Pulmonary Disease Patients (InformedTogether): Feasibility Study
Source: J Particip Med. 2018 May 14;10(2):e7. doi: 10.2196/jopm.9877 (PMC7251980; doi:10.2196/jopm.9877)
Supplement: Multimedia Appendix 5 [file jopm_v10i2e7_app5.pdf]

MA5: Distress experienced while viewing decision aid

Distress Scenario	Sample Text from Clinician Patient Encounter	Significant patient characteristics
Patient expresses distress, and asks to stop viewing decision aid	**Clinician participant: If you feel that this is too stressful for you or you're getting too anxious, we can certainly stop. You tell me what you want to do.**<br>Patient participant: It's a lot to take. I feel I've had enough.	Patient: Severe COPD<br>Deceased by time of 1-month follow up
Patient expresses distress but continues after clinician uses languages acknowledging the difficulty of conversation	**Clinician participant: So in the next few slides, if you would like, we can talk about the upside and downside of breathing tubes, so you can make a decision that's right for you. Do you want to go on?**<br>Patient participant: I don't know if I want to go on.<br>**Clinician participant: No? Okay. You don't want to talk about it?**<br>Patient participant: No, because I don't feel comfortable yet about talking about this. Because I really wasn't expecting all this.<br>**Clinician participant: Yeah, of course, and I realize this conversation—the whole point of this, again, is because it's a sensitive topic, and realistically this is—a lot of people aren't informed. They don't know what the difference is. Yeah, it's not specifically for—**<br>Patient participant: No, no, I hear.<br>**Clinician participant: So I realize this conversation might be making you anxious or upset, but I want—before you go on, I want to let you know that's normal to feel anxiety or to be afraid of thinking—becoming very sick, so I don't mean to upset you, but I want to make some time to make sure that you and your family are prepared. The best choice is—**<br>Patient participant: This is stuff that we never discuss.....<br>**Clinician participant: Okay? Should we continue? It's up to you. I mean…**<br>Patient participant: Go ahead. Let's see.	Patient: severe COPD,<br>Never considered decision before
Patient expresses distress at thought of having her children seeing her intubated	Patient participant: I don't want to see my kids…they would suffer if they saw me going through something like that.<br>**Clinician participant: Going through—yeah, so that's why the topic itself is so sensitive.**<br>Patient participant: You know the people that are being left behind or what have you, the ones that you love, you don't—because I know how they felt when my husband was taking his last breath, and I had to make the decision for them to turn it off, but I held on as long as I could for their sake because, you know, it was a hard thing, but somebody has to make the decision…<br>**Clinician participant: So let's review what you learned so far, so in your own words, can you summarize what you learned so far about intubation and comfort measures? Like, for example, what would you say to your family about what you've seen today?**<br>Patient participant: You know, my husband was sick for many years, and I was back and forth to [the hospital] from 2003 back and forth until he died in 2012. I myself would not want to put my kids through going to the hospital every day, watching me slowly but surely just…I don't want—my body couldn't take it because I couldn't take watching them see it. I wouldn't want to stay here. I…I…I just don't wanna.	Patient: COPD severe<br>Experienced having to remove her husband from life support
