# Supplementary material for: A Decision Aid to Support Shared Decision Making About Mechanical Ventilation in Severe Chronic Obstructive Pulmonary Disease Patients (InformedTogether): Feasibility Study
Source: J Particip Med. 2018 May 14;10(2):e7. doi: 10.2196/jopm.9877 (PMC7251980; doi:10.2196/jopm.9877)
Supplement: Multimedia Appendix 6 [file jopm_v10i2e7_app6.pdf]

MA6: Patient Interest in Trial Intubation

**Context for discussion of trial intubation**	**Sample Text from Clinician Patient Encounter**
**Patient's evolution toward trial intubation from clinic visit to 1-month follow up interview**	<u>During Clinic Visit:</u><br>**Clinician: Have you ever given any, I mean, as someone who has seen that in your own mother, and knows that—I mean, have you ever given any thought to if your COPD, if you got worse, if you would want to be put on a machine or—**<br>Patient: The way I rationalize that, like, no. No machines, you know.<br><br><u>Same patient at 1-month follow-up :</u><br>**Interviewer: Did you see the part where you have those things that you can kind of, scroll—it is called the "values clarification exercises" where it looks at what is most important to you and you can use the sliding scale?**<br>Patient: Oh yeah, I did that.<br>**Interviewer: Okay. Was that helpful?**<br>Patient: Very, yeah.<br>**Interviewer: Okay. So if you were to have a bad exacerbation, you would want to be treated with a breathing machine, at least, to try it?**<br>Patient: Uh, yeah… I think other questions that I had the…I'm trying to…I think the most important question for me was…about the breathing machine could I have an Advance Directive indicating timeframe that I want to be put on a breathing machine. For example, like, ten days…(or something?), like, two weeks, [unintelligible]. You can do that right?<br>**Interviewer: Like a trial with it?**<br>Patient: For example, my daughter would communicate my wishes if I need to go on the breathing machine, I would do so, but not more than one month [unintelligible], you know?<br>**Interviewer: Right, so you wouldn't want to be on it for a year, you would say, give it a month and then that is it?**<br>Patient: Right.<br>**Interviewer: So, did your decision change since you saw this information with the doctor?**<br>Patient: Absolutely, because I really hadn't thought about it in depth, you know.
**Patient bringing up trial intubation while viewing the decision aid with clinician**	Patient participant: "Is it a permanent thing, this breathing tube? Is there some kind of form or consent form, or something that shows like a proxy-type thing, that says, this is what I want unless you feel like I will be a vegetable, or I won't have any quality of life?"<br>**Clinician participant: Right. And part of the beauty of this new form is, it's, "We want this, we want this, and I want a trial of this. And if it looks like it's not working, then make me comfortable." It is that we don't necessarily have to pull the proverbial plug, we can kind of see how you do…"**
**Patient discussing trial intubation with researcher during 1-month follow up interview**	**Interviewer: If you were to have a bad COPD exacerbation would you want to be treated with the breathing machine?**<br>Patient participant: Yes.<br>**Interviewer: Okay. And has this decision changed since your conversation with your doctor at that visit? In other words, from seeing the decision aid?**<br>Patient participant: It's like I can be on the breathing machine, but if I feel that it's not good, or it hurts too much, it's not a good quality of life, or I can make—you know, I can make the decision to come off of it. So, you know, you're kind of open….<br>**Interviewer: Right.**<br>Patient participant: You know? So, it really is okay to start and see how it goes with that. I think that that's important to know. That you can start with it. If you feel that you don't want to do it, this is not how you want to live, you know, while you're on the breathing machine, you know, discuss it with a family member or somebody close to you and say, "listen I really…I can't do this." And more on a factual level than emotional level that it was…it's good that I have the choice. So, I'd definitely do it to start. And that was the decision I came to after meeting with you and Dr. [name].<br>**Interviewer: Okay, so yours is that you would—before that you really didn't have that decision in place?**<br>Patient participant: No. I wasn't sure.
