# Supplementary material for: A Decision Aid to Support Shared Decision Making About Mechanical Ventilation in Severe Chronic Obstructive Pulmonary Disease Patients (InformedTogether): Feasibility Study
Source: J Particip Med. 2018 May 14;10(2):e7. doi: 10.2196/jopm.9877 (PMC7251980; doi:10.2196/jopm.9877)
Supplement: Multimedia Appendix 7 [file jopm_v10i2e7_app7.pdf]

MA7: Univariable Analysis: Associations between Communication and Outcomes

	**SatCom1-10**	**SatCom11-20**
**Change in knowledge**	-0.08 (p 0.66)	-0.14 (p 0.41)
**Change in motivation**	-0.08 (p 0.66)	-0.08 (p 0.65)
**DCS post**	-0.29 (p 0.09)	-0.46 (**p 0.005**)
**Change in DCS**	-0.01 (p 0.95)	0.11 (p 0.62)
**OPTION score**	0.23 (p 0.18)	0.28 (p 0.09)
Results represent Spearman's correlation coefficients and the corresponding p-values		
