# Supplementary material for: A Decision Aid to Support Shared Decision Making About Mechanical Ventilation in Severe Chronic Obstructive Pulmonary Disease Patients (InformedTogether): Feasibility Study
Source: J Particip Med. 2018 May 14;10(2):e7. doi: 10.2196/jopm.9877 (PMC7251980; doi:10.2196/jopm.9877)
Supplement: Multimedia Appendix 8 [file jopm_v10i2e7_app8.docx]

**MA8: Univariable Analysis: Associations between Outcomes and Patient Demographics**

| **Outcomes** | **Age**  **(34-60;**  **61-75; >75)** | **Marital Status (yes/no)** | **Sex** | **Ethnicity (Hispanic/**  **Black/**  **White)** | **Religion (yes/no)** | **Education level**  **(5 levels)** | **Economic Class**  **(5 levels)** | **English Fluent (yes/no)** | **Numeracy (high vs low)** |
| --- | --- | --- | --- | --- | --- | --- | --- | --- | --- |
| **Change in knowledge** | 5.4 (p 0.07) | 4.7 (p 0.32) | 0.6 (p 0.46) | 1.3 (p 0.51) | 0.1 (p 0.77) | 9.1 **(p 0.05)** | 4.3 (p 0.37) | 0.2 (p 0.67) | 2.0 (p 0.16) |
| **Change in motivation** | 0.5 (p 0.79) | 3.3 (p 0.50) | 2.7 (p 0.1) | 1.4 (p 0.49) | 1.0 (p 0.31) | 6.1 (p 0.19) | 0.4 (p 0.98) | 0.5  (p 0.5) | 0.5  (p 0.56) |
| **Change in motivation**  **at 1 month** | 0.7 (p 0.71) | 6.0 (p 0.19) | 0.4 (p 0.52) | 3.0 (p 0.22) | 1.7 (p 0.19) | 5.2 (p 0.27) | 3.8 (p 0.43) | 0.5  (p 0.48) | 0.1  (p 0.75) |
| **DCS post** | 2.0 (p 0.37) | 6.4 (p 0.17) | 0.0 (p 0.93) | 3.7 (p 0.16) | 2.4 (p 0.12) | 8.1 (p 0.09) | 3.9 (p 0.53) | 1.9  (p 0.17) | 2.1  (p 0.14) |
| **Change in**  **DCS** | 0.6 (p 0.74) | 0.8(p 0.94) | 0.0 (p 0.97) | 3.8 (p 0.15) | 3.9 **(p 0.05)** | 5.3 (p 0.25) | 1.8 (p 0.77) | 1.3  (p 0.25) | 0.4  (p 0.55) |
| Results from Nonparametric Kruskal-Wallis test with the corresponding p-values | | | | | | | | | |
