# Supplementary material for: A Decision Aid to Support Shared Decision Making About Mechanical Ventilation in Severe Chronic Obstructive Pulmonary Disease Patients (InformedTogether): Feasibility Study
Source: J Particip Med. 2018 May 14;10(2):e7. doi: 10.2196/jopm.9877 (PMC7251980; doi:10.2196/jopm.9877)
Supplement: Multimedia Appendix 9 [file jopm_v10i2e7_app9.pdf]

MA9: Univariable Analysis: Associations between Outcomes and Patient Self-Rated Health

Outcomes	Self-rated health (5 levels)	Self-rated QOL (1-5 vs 6-10)	Self-rated COPD level	Hospitalizations in past yr (<=2 vs >2)
**Change in knowledge**	6.42(p 0.17)	6.1 **(p 0.02)**	6.2 (p 0.19)	1.1 (p 0.30)
**Change in motivation**	3.4 (p 0.49)	0.0 (p 0.96)	5.0(p 0.29)	2.2 (p 0.14)
**Change in motivation at 1 month**	5.7 (p 0.22)	6.3 **(p 0.01)**	2.7(p 0.6)	5.1 (p 0.02)
**DCS post**	7.2 (p 0.13)	6.0 **(p 0.02)**	3.1 (p 0.55)	1.8 (p 0.18)
**Change in DCS**	0.4 (p 0.98)	1.8 (p 0.18)	1.5 (p 0.67)	0.3 (p 0.59)

Results from Nonparametric Kruskal-Wallis test with the corresponding p-values
